# Planar Hall effect from the surface of topological insulators

**DOI:** 10.1038/s41467-017-01474-8

**Published:** 2017-11-07

**Authors:** A. A. Taskin, Henry F. Legg, Fan Yang, Satoshi Sasaki, Yasushi Kanai, Kazuhiko Matsumoto, Achim Rosch, Yoichi Ando

**Affiliations:** 1 Physics Institute II, University of Cologne, Zülpicher Str. 77, 50937 Köln, Germany; 2 Institute for Theoretical Physics, University of Cologne, Zülpicher Str. 77, 50937 Köln, Germany; 3 Institute of Scientific and Industrial Research, Osaka University, Mihogaoka 8-1, Ibaraki, Osaka, 567-0047 Japan; 4 Present Address: School of Physics and Astronomy, University of Leeds, Leeds, LS2 9JT UK

## Abstract

A prominent feature of topological insulators (TIs) is the surface states comprising of spin-nondegenerate massless Dirac fermions. Recent technical advances have made it possible to address the surface transport properties of TI thin films by tuning the Fermi levels of both top and bottom surfaces. Here we report our discovery of a novel planar Hall effect (PHE) from the TI surface, which results from a hitherto-unknown resistivity anisotropy induced by an in-plane magnetic field. This effect is observed in dual-gated devices of bulk-insulating Bi_2−*x*_Sb_*x*_Te_3_ thin films, where the field-induced anisotropy presents a strong dependence on the gate voltage with a characteristic two-peak structure near the Dirac point. The origin of PHE is the peculiar time-reversal-breaking effect of an in-plane magnetic field, which anisotropically lifts the protection of surface Dirac fermions from backscattering. The observed PHE provides a useful tool to analyze and manipulate the topological protection of the TI surface.

## Abstract (web summary)

Topological surface states can lose their protection in many ways but the subtle mechanisms remain far from well understood. Here, Taskin et al. report a novel planar Hall effect in dual-gated Bi_2−*x*_Sb_*x*_Te_3_ thin films, originating from anisotropic lifting of time reversal symmetry protection by an in-plane magnetic field.

## Introduction

The two-dimensional (2D) Dirac fermions on the surface of topological insulators (TIs)^1–3^ are immune to localization by a random scalar potential^4^ and are often said to be topologically protected. Besides the bulk-edge correspondence of a topological system to guarantee the gapless nature^3^, there are two closely related reasons for this protection: First, the *π* Berry phase associated with massless Dirac fermions protects them from weak localization effect^5^. Second, the spin is perpendicularly locked to the momentum, which suppresses the backscattering on non-magnetic scatterers^1–3^. However, the topological protection can be lifted in several situations. For example, when a sample is too thin and the wavefunctions of the top and bottom surface states overlap, the hybridization between the two opens up a gap at the Dirac point^6^, leading to a loss of topological protection^7^. Also, since time-reversal symmetry (TRS) is the prerequisite of topological states in TIs^1–3^, breaking of TRS is another way to lift the topological protection. Applying a magnetic field perpendicular to the TI surface introduces a mass term in the Dirac Hamiltonian to open up a gap in the surface states. When the field is applied along the surface of a TI, TRS is also broken, but such a parallel magnetic field will not affect helical surface states besides a shift of the Dirac dispersion in the momentum space; hence, no gap will open in the Dirac dispersion for high-symmetry orientations of the surface and the magnetic field (see Supplementary Note 1 for details).

Our experiment is designed to address the effect of the parallel magnetic field on the surface transport, when TRS is broken, but the massless Dirac state is preserved. We found that the scattering of Dirac fermions in this situation becomes anisotropic because the spin-momentum locking causes a difference in the scattering amplitudes for particles with the spin parallel and perpendicular to the magnetic field direction. This leads to a magnetic field-induced anisotropy in the resistivity measured along and perpendicular to the field, which results in a novel planar Hall effect (PHE). In other words, this intriguing effect is a manifestation of the momentum-selective lifting of the topological protection due to TRS breaking.

## Results

### Dual-gate device

To access the surface transport properties, one needs to suppress the bulk contribution in the total conductance. There are several ways to achieve this:^8–12^ The most effective one is the compensation of donors and acceptors in the TI material to bring the Fermi level into the bulk band gap. Reducing the thickness of a sample can also be effective, due to a reduced bulk/surface ratio. At present, thin-film samples of TIs grown by the molecular beam epitaxy (MBE) technique are among the best for surface transport experiments^13–16^. For example, Bi_2−*x*_Sb_*x*_Te_3_ (BST) thin films, in which the optimization of the composition can give almost perfect compensation, were used for studying the integer quantum Hall effect on the TI surface^17^.

**Fig. 1:**
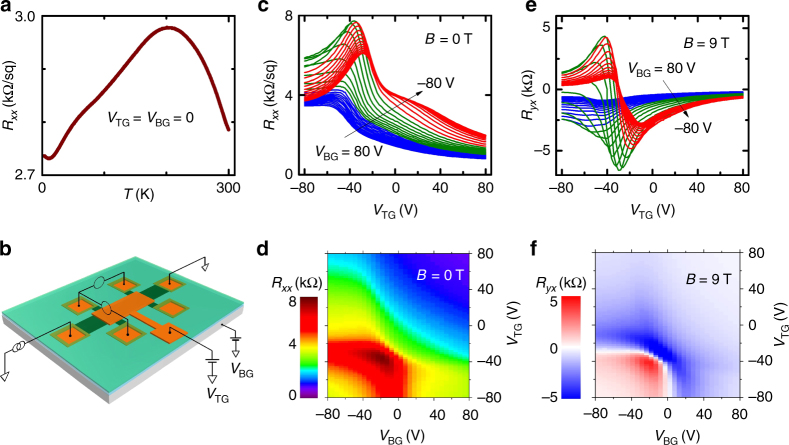
Dual-gating of BST films. **a** Temperature dependence of *R*_*xx*_ in a 17-nm-thick device at zero gate voltages (*V*_TG_ = *V*_BG_ = 0). **b** Schematics of the dual-gate Hall-bar device and the measurement configuration. **c**, **d** Gate–voltage dependencies of *R*_*xx*_ in 0 T at 2 K. **e**, **f** Gate–voltage dependencies of *R*_*yx*_ in the perpendicular magnetic field of 9 T at 2 K

For the present experiments, BST films with a bulk-insulating composition (*x* ≈ 1.7) were grown on sapphire by MBE. A typical temperature dependence of the sheet resistance *R*_*xx*_ in a bulk-insulating sample is shown in Fig. 1a. Below about 200 K, the resistivity is dominated by metallic surface transport. The magnitude of *R*_*xx*_ depends on the charge carrier density *n*_*s*_ on the top and bottom surfaces of the film, which can be controlled by electrostatic gating^18^. Our dual-gate device, shown schematically in Fig. 1b, provides the ability to tune *n*_*s*_ on both surfaces. For example, as shown in Fig. 1c, *R*_*xx*_ can reach a high value of ~8 kΩ by suitably tuning the top- and bottom-gate voltages, *V*_TG_ and *V*_BG_, respectively. The effect of this dual-gating can be clearly seen in the color mapping shown in Fig. 1d, where the maximum in *R*_*xx*_(*V*_TG_, *V*_BG_), corresponding to the dark-red region, signifies the simultaneous crossing of the Dirac points on both top and bottom surfaces. The Hall resistance *R*_*yx*_ was measured in magnetic fields perpendicular to the films, and its gate–voltage dependencies are shown in Fig. 1e for *B* = 9 T; here, one can see a sharp change between *n*- and *p*-type carriers in a specific range of gate voltages. The zero-crossing of *R*_*yx*_, which can be easily recognized in the color mapping shown in Fig. 1f as a white band separating red (*p*-type) and blue (*n*-type) regions, can be used as an indicator of the Dirac-point crossing of the Fermi level.

### Planar Hall effect

**Fig. 2:**
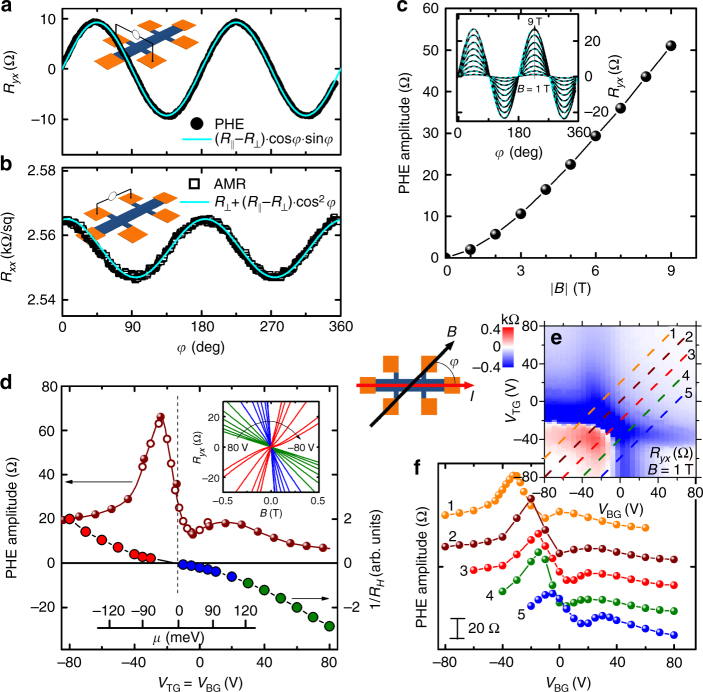
Planar Hall effect. **a** Angular dependence of the planar *R*_*yx*_ data at *V*_TG_ = *V*_BG_ = 5 V measured at 2 K in the magnetic field of 9 T rotated in the film plane (inset shows the configuration); blue solid line is a fit to (${R_\parallel } - {R_ \bot }$)cos*φ*sin*φ*, where *φ* is defined in the central inset. **b** Angular dependence of *R*_*xx*_ in the same conditions as in **a**; blue solid line is a fit to (${R_\parallel } - {R_ \bot }$)cos^2^ *φ*. **c** Magnetic-field dependence of the PHE amplitude ($\equiv {R_\parallel } - {R_ \bot }$) at *V*_TG_ = −80 V and *V*_BG_ = 80 V; inset shows the raw *R*_*yx*_(*φ*) data and their fits in various *B*. **d** Gate–voltage dependence of the PHE amplitude for *V*_TG_ = *V*_BG_ in the in-plane 9-T field (left axis) and the effective total carrier density (right axis), obtained from the low-field Hall data (shown in the inset); vertical dashed line marks the Dirac-point crossing, and the scale-bar inset depicts the estimated change of the Fermi level. **e** Color mapping of *R*_*yx*_(*V*_TG_,*V*_BG_) measured in the out-of-plane 1-T field, on which different dual-gating paths for the PHE-amplitudes measured in the in-plane 9-T field shown in **f** are indicated; curves in **f** are shifted vertically for clarity

Our main result, observation of the PHE, is shown in Fig. 2. For these measurements, the magnetic field was applied parallel to the film and was rotated within the film plane. The angle *φ* between the field and the current direction is defined in the central inset of Fig. 2. The planar Hall resistance *R*_*yx*_, i.e., the transverse resistance measured across the width of the sample perpendicular to the current (as shown in the inset of Fig. 2a), shows a non-zero value for all field directions except for the parallel and perpendicular orientations. In fact, it follows the ~ cos*φ* sin*φ* angular dependence as exemplified in Fig. 2a for *V*_TG_ = *V*_BG_ = 5 V and *B* = 9 T. Such a behavior is normally not expected for non-magnetic materials. The 180°-periodic angular dependence was also observed in the longitudinal resistance *R*_*xx*_ as shown in Fig. 2b; this kind of resistivity oscillation is generally called anisotropic magnetoresistance (AMR). The observed AMR follows the ~cos^2^ *φ* angular dependence. Phenomenologically, both PHE and AMR stem from an anisotropy in the resistance tensor, and the observed *φ* dependence is expected when the magnetic field sets the anisotropy axis, along which the resistance becomes larger (see Supplementary Note 2 for details).

According to the resistance-tensor phenomenology, the amplitudes of PHE and AMR should be the same and are both written as ${R_\parallel } - {R_ \bot }$, where ${R_\parallel }$ (*R*_⊥_) is the sheet resistance for $B\parallel I$ (*B* ⊥ *I*). Nevertheless, due to a possible misalignment of the experimental plane of rotation with respect to the film plane, the observed AMR can be contaminated by the contribution from the ordinary orbital magnetoresistance $\Delta R_ \bot ^*$, which comes from a finite magnetic field component $B_ \bot ^*$ perpendicular to the film (see Supplementary Note 3 for details). Although $B \bot I$ is normally very small, a large surface sheet resistance (up to several kΩ) can cause the contribution from the orbital MR to become comparable to the amplitude of the AMR (~ several tens of Ω). To make matters worse, upon the magnetic field rotation, $B_ \bot ^*$ will change as ~cos*ϕ*, which, combined with the $\Delta R_ \bot ^*\sim {B^2}$ behavior expected for the orbital MR, causes the spurious signal to present a ~cos^2^ *φ* dependence; this is virtually indistinguishable from the genuine AMR signal, and hence the amplitudes of the AMR in actual experiments are not always reliable (see Supplementary Note 4 for details). On the other hand, the ordinary Hall contribution due to $B_ \bot ^*$ is antisymmetric with respect to *B* and can be easily removed from the PHE signal by taking the data in both positive and negative *B*. Therefore, PHE gives the genuine amplitude of ${R_\parallel } - {R_ \bot }$, and all quantitative discussions in this paper are based on the measurements of PHE. Figure 2c shows an example of the magnetic-field dependence of ${R_\parallel } - {R_ \bot }$, which is super-linear up to 9 T and shows no sign of saturation.

In ferromagnets, the phenomenon of AMR has been known for a long time^19^; in fact, Lord Kelvin reported the AMR almost 200 years ago. The understanding of its origin was eventually established through works of Mott (1936)^20^, Smit (1951)^21^, Campbell, Fert, and Jaoul^22^, and extended by others. This effect is best understood in diluted magnetic alloys, where the coexistence of *s*- and *d*-bands near the Fermi energy and a strong spin-orbit coupling are the two main ingredients for the AMR. The rotation of the magnetization by the magnetic field changes the population of unoccupied *d*-states with respect to the current direction, leading to a change in the scattering rate between *s*- and *d*-bands. Clearly, this mechanism is not applicable to a non-magnetic TI investigated here (see Supplementary Note 5 for details). Trushin et al.^23^ argued that the scattering from polarized magnetic impurities contributes to AMR in Rashba systems with spin-split Fermi surfaces, an effect which may have been observed in a 2D electron system at the LaAlO_3_/SrTiO_3_ interface^24^. Note that at the LaAlO_3_/SrTiO_3_ interface, even though it is nominally non-magnetic, 3*d* Ti orbitals undergo crystal-field-induced splitting with some preferential filling to give rise to magnetism^24^, which is required in the theory of Trusin et al.^23^.

### Gate–voltage dependence of the PHE amplitude

To address the origin of the PHE and AMR in TI films, we took advantage of our dual-gate capability to tune the density and the type of carriers (and hence their helicity) on both surfaces, to see how these parameters influence the observed anisotropy. It turns out that for both *n*- and *p*-type states, the anisotropy is always positive, i.e., ${R_\parallel }  >{R_ \bot }$. Moreover, when the Fermi level is moved through the Dirac point and the surface conduction is changed from *n*- to *p*-type, the PHE amplitude was found to present an unusual two-peak structure with a local minimum at the Fermi-level position close to the Dirac point. Figure 2d shows an example of the PHE amplitude vs. gate voltage along the dual-gating path with *V*_TG_ = *V*_BG_, presenting two peaks and a minimum; this minimum is located near the gate voltage, where the effective total carrier density (deduced from the low-field Hall coefficient *R*_*H*_) becomes zero, which roughly corresponds to the Dirac-point crossing (see Supplementary Notes 6 and 7 for details). Measurements along different dual-gating paths near the Dirac point indicated in Fig. 2e found essentially the same behavior, apart from a slight broadening and a shift along the *V*_*BG*_-axis related to the shift in the transition from the *n*- to *p*-type region; this means that the characteristic two-peak structure is always associated with the Dirac-point crossing.

This result is in stark contrast to the result of the AMR measurements in exfoliated flakes of another compensated TI material, BiSbTeSe_2_^25^, where the AMR amplitude was observed to change from positive to negative upon applying a gate voltage to a 160-nm-thick flake from a bottom gate and measuring *R*_*xx*_ on the top surface. In this regard, we were able to reproduce similar behavior in our devices by intentionally setting a misalignment angle of ~ 1° upon rotation. In our series of control experiments, including simultaneous measurements of AMR and PHE and adoptions of different mounting configurations (see Supplementary Note 4 for details), we found that the negative AMR in our device is an artifact due to the orbital MR and is strongly gate–voltage dependent (see Supplementary Figs. 3 and 4). This conclusion is also supported by the temperature dependences of AMR and PHE: their amplitudes merge at high temperature where the orbital MR amplitude diminishes (see Supplementary Fig. 6). We note that the bulk contribution could also play a role in the negative AMR in a thick TI flake, because the longitudinal magnetoresistance can become negative in the bulk transport, as recently reported for Bi_2_Se_3_ films^26^.

### Origin of the PHE

The occurrence and sign of the observed PHE/AMR follows directly from the spin-momentum locking of Dirac fermions on the TI surface and the associated topological protection from backscattering in the absence of broken TRS, which can be lifted by an in-plane magnetic field. The backscattering is highly sensitive to the relative orientation of this field and the electron velocity, as is shown in the following theoretical calculations.

When describing the PHE/AMR theoretically, one first notes that an in-plane and uniform magnetic field has no effect on the electrons if one models the surface based on the 2D Dirac equation and potential scattering from disorder: orbital effects are absent and the Zeeman coupling can be gauged away by a simple shift of the Dirac point^27,28^. In reality, however, the uniform magnetic field in a disordered medium with spin-orbit interactions will generate magnetic fields at random positions that can induce spin-flip scattering. To describe this effect, we consider a two-dimensional model where the Dirac electrons hybridize with impurities located at random positions **R**_*i*_ with the density *n* ^imp^,

1 $$H = \mathop {\sum}\limits_{{\bf{k}},\alpha ,\beta } {{h_{\alpha \beta }}({\bf{k}})\psi _\alpha ^\dag ({\bf{k}}){\psi _\beta }({\bf{k}})} + \mathop {\sum}\limits_{\alpha ,\beta } {\left( {\left( {\epsilon - \mu } \right){\delta _{\alpha \beta }} - {\bf{B}} \cdot {\bf{\sigma }}} \right){{d}}_\alpha ^\dag {{{d}}_\beta }} \\ + V\mathop {\sum}\limits_{{\bf{k}},\alpha ,i} {{e^{ - i{\rm{k}} \cdot {{\bf{R}}_i}}}\psi _\alpha ^\dag ({\bf{k}}){{{d}}_\alpha }} + {\rm{h}}.{\rm{c}}.$$

Here the first term accounts for the motion of the surface Dirac fermions: $\hbar {v_{\rm{F}}}{({k_{\it{x}}}{\sigma _{\it{y}}} - {k_{\it{y}}}{\sigma _{\it{x}}})_{\alpha \beta }} - \mu {\delta _{\alpha \beta }}$, where *μ* is the electrochemical potential controlled by the gate voltage. The second term describes a localized impurity state with resonance energy ϵ, which, in the third term, hybridizes with the continuum states with hybridization strength *V*. The magnetic field has already been gauged away leaving the only remaining effect as the Zeeman coupling to the impurity states. Here we use units where *g* ^imp^ *μ*_B_/2 = 1, where *g* ^imp^ is the *g* factor of the impurity. While our model is not expected to give a microscopic description of the actual disorder in our experiment, it is a minimal model capturing the field-induced anisotropic scattering and the interplay of magnetic (spin-flip) and non-magnetic (non-spin-flip) scattering essential to explain the experiment.

To calculate the conductivity *σ*, we employ a self-consistent T-matrix calculation^29,30^, valid in the limit of small *n* ^imp^ for arbitrary values of *V* and ϵ (see Supplementary Note 8 for details). While this approximation does not treat correctly all logarithmic corrections, it is known^30^ to give accurate results in situations like our experiment where weak localization or antilocalization effects are not visible. To obtain conductivities within the T-matrix approximation quantitatively, we have to include the corresponding vertex corrections (see Supplementary Note 9 for details), which enhance the conductivity away from the Dirac node by approximately a factor of 2 (similar vertex corrections vanish for local scattering in graphene)^30^. For the dimensionless ratio, $\delta (\mu ) = \frac{{{\sigma _ \bot }(\mu ) - {\sigma _\parallel }(\mu )}}{{{\sigma _\parallel }(\mu )}}$, we find, however, that vertex corrections have only a minor effect.

**Fig. 3:**
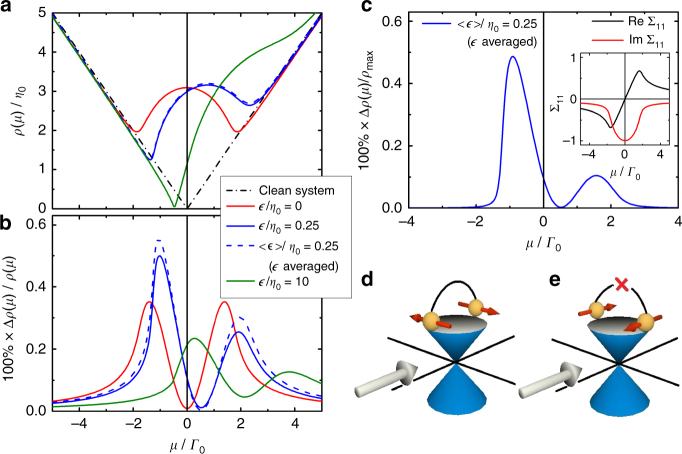
Origin of MR anisotropy. **a** Local density of states for randomly distributed impurities of concentration *n* ^imp^ = 0.005 as a function of normalized chemical potential *μ*/*Γ*_0_ for three different impurity-resonance energies ϵ (*solid lines*) and for a Gaussian distribution of ϵ with width ${\eta _0} \equiv {V^2}{{\Gamma} _0}/4\pi v_{\rm{F}}^2$ centred at ϵ/*η*_0_ = 2.5 (blue dashed line). **b** The dimensionless ratio $\delta (\mu ) = \frac{{{\sigma _ \bot }(\mu ) - {\sigma _\parallel }(\mu )}}{{{\sigma _\parallel }(\mu )}} = \frac{{{\rho _\parallel }(\mu ) - {\rho _ \bot }(\mu )}}{{{\rho _ \bot }(\mu )}}$ (in percent) as a function of *μ* for different ϵ (same as in **a**), showing a characteristic two-peak structure near the Dirac point. **c** The normalized anisotropy magnitude Δ*ρ*(*μ*)/*ρ*_m*ax*_ (in percent) calculated for the Gaussian distribution of ϵ; this quantity is more appropriate than *δ*(*μ*) for comparison with experiment, where bulk contributions will be present in *ρ*(*μ*). Inset shows the real and imaginary parts of the diagonal self-energy matrix element Σ_11_ for unbroken particle–hole symmetry (ϵ = 0). **d**, **e** Schematic picture of the scattering on spin-polarized impurities. **d** For Dirac fermions with spins perpendicular to the magnetic field (gray arrow), spin-flip scattering is allowed due to broken TRS. **e** For Dirac fermions with spins parallel/anti-parallel to the field, spin-flip scattering remains prohibited

The main result of our calculation is shown in Fig. 3b, where *δ*(*μ*) is shown as a function of the chemical potential for various values of ϵ. As in the experiment a clear two-peak structure emerges. The asymmetry of the two peaks is controlled by ϵ parametrizing the breaking of particle–hole symmetry by our scatterers^31–33^. The peaks track precisely the minima in the local density of states shown in Fig. 3a.

Whilst we include vertex corrections in our calculation, it is instructive to consider a simplified version ignoring vertex corrections, considering only the contribution from the product of retarded and advanced Green’s function^30^. For *T* = 0 and field ***B*** we obtain

2 $${\sigma ^{\parallel / \bot }} =  \frac{{2{e^2}v_{\rm{F}}^2}}{\pi }{\int} {\frac{{{{\rm{d}}^2}{\bf{k}}}}{{{{(2\pi )}^2}}}\frac{{{{\left| {\mu - {\Sigma _{11}}} \right|}^2} \pm \left( {v_{\rm{F}}^2k_\parallel ^2 - {{\left| {{v_{\rm{F}}}{k_ \bot } - {\Sigma _{12}}} \right|}^2}} \right)}}{{{{\left| {{{\left( {\mu - {\Sigma _{11}}} \right)}^2} - \left( {v_{\rm{F}}^2k_\parallel ^2 + {{\left( {{v_{\rm{F}}}{k_ \bot } - {\Sigma _{12}}} \right)}^2}} \right)} \right|}^2}}}} \\ \approx  {{{\sigma _0}\left( {1 \mp \frac{c}{2}{{\left( {\frac{{{\rm{Im}}{\Sigma _{12}}}}{{{\rm{Im}}{\Sigma _{11}}}}} \right)}^2}} \right),}}\hskip10.4pc$$

where Σ_11_ and Σ_12_ are the diagonal (non-spin-flip) and off-diagonal (spin-flip) contributions to the self-energy at *μ*, respectively; the prefactor *c* is numerically found to be 0.8–1.2, depending on parameters. The second equality was derived by using ${\Sigma _{12}} \ll {\Sigma _{11}}$ and adsorbing the real part of Σ_12_ by a shift of *k*_y_. The expression of *σ* in Eq. (2) reproduces the expected behavior: The conductivity parallel to the magnetic field is reduced, as backscattering in parallel direction is activated by the field^23^, as shown schematically in Fig. 3d–e. The effect is quadratic in *B* for small *B* as ImΣ_12_, the spin-flip scattering rate, is linear in *B* for small *B*.

## Discussion

According to Eq. (2), the peaks in the anisotropic resistivity arise from peaks in ImΣ_12_. As shown in the Supplementary Note 8, ImΣ_12_ ∝ Im[(Σ_11_)^2^] = 2ImΣ_11_ReΣ_11_. Using this result, we find that the origin of the peak can be traced back to peaks in ReΣ_11_. Due to the Kramers–Krönig relation, these peaks occur when |ImΣ_11_| quickly diminishes as *μ* moves away from the Dirac point (see Fig. 3c inset); this diminishment occurs roughly at $\mu \sim \pm {\Gamma} _0$, where *Γ*_0_ = −ImΣ_11_(*μ* = 0) is the non-spin-flip scattering rate at the Dirac point. Hence, the location of *μ* associated with a peak gives a measure of Γ_0_. Estimating *μ* from the Hall data assuming a Fermi velocity of 3.9 × 10^5^ m/s^34,35^ (see Fig. 2d inset), we obtain for our sample ${{\Gamma} _0}\sim 50{\kern 1pt}$ meV, corresponding to a mean free path *v*_F_/*Γ*_0_ of ~ 70 Å.

Furthermore, according to Eq. (2), the amplitude of the anisotropy is set by the square of the ratio of spin-flip and non-spin-flip scattering rates. Since our data show $\delta \sim 1\%$ at 9 T, one may infer that about 10% of the scattering processes are spin-flipping at 9 T and the corresponding spin-flip scattering rate and mean free path are ~ 5 meV and ~ 700 Å, respectively. For a system without magnetic impurities, this spin-flip scattering rate is remarkably high. As the magnetization of non-magnetic impurities is governed by the ratio of Zeeman splitting and hybridization strength, the large *g* factor typical for Bi-based topological insulators and the low-density of states of Dirac systems, which strongly suppresses the hybridization, conspire to enhance magnetic scattering.

It is important to note that the two-peak structure in the anisotropy and its relation to the microscopic parameters are found to be robust even when we consider distributions of *V* and ϵ. As an example, Fig. 3b compares the results for the cases when ϵ is fixed or has a Gaussian distribution (the result is similar for a Gaussian distribution of *V* as shown in Supplementary Note 9). Note, however, that microscopic details will strongly affect the asymmetry of the peaks. The experimental data for the PHE amplitude (Fig. 2d) is best compared to Fig. 3c.

The proposed theoretical model gives a clear physical picture for the origin of the MR anisotropy in the TI surface; namely, the spin-momentum locking protects the surface Dirac fermions from backscattering in zero field, but the in-plane magnetic field breaks TRS and spin-polarizes randomly distributed impurities, drastically changing this topological protection. For spins perpendicular to the field, the protection is lifted and backscattering is allowed due to TRS breaking as schematically shown in Fig. 3d. In contrast, for spins parallel or anti-parallel to the field [shown in Fig. 3e], backscattering is still forbidden. This anisotropy in the scattering rate directly results in the AMR and PHE: For the field direction parallel to the current (and hence perpendicular to the spin orientation), the allowed backscattering results in the increased resistance *R*_||_, while for the field direction perpendicular to the current, the resistance *R*_⊥_ will be much less affected. This is the reason for a positive anisotropy amplitude (i.e. *R*_||_−*R*_⊥_ ≥ 0). We further show that, from the two-peak structure of the anisotropy, one can infer the effective scattering rates for both spin-flip and non-spin-flip scatterings. Therefore, the PHE discovered here represents a novel signature of TRS breaking in TIs and provides microscopic information on the topological surface transport.

## Methods

### MBE growth of high-quality BST films

Bi_2−*x*_Sb_*x*_Te_3_ films with the thickness in the range of 11–17 nm were grown on sapphire (0001) substrates by co-evaporation of high-purity Bi, Sb, and Te from Knudsen cells in the ultra-high vacuum MBE chamber. The flux ratio of Bi and Sb was optimized for obtaining most bulk-insulating films and was kept at 1:5.5. The Te flux exceeded the aggregated flux of Bi and Sb by at least 10 times. The deposition was done in three temperature steps: at 230 °C for 5 min, at 280 °C for 5 min, and finally at 325 °C for a time period which is sufficient to grow a film with a desirable thickness. The thickness and morphology of the grown films were measured ex situ by AFM.

### Device microfabrication

To make a dual-gate device, the grown films need to be transferred from the sapphire substrate to a Si/SiO_2_ wafer, which serves as a back-gate electrode and dielectric (SiO_2_ is 150-nm thick). The separation of a BST film from the substrate was done by first spin-coating the film with PPMA and then dipping into 5% KOH aqueous solution to initiate the detachment. The full detachment was done by slowly dipping the film into distilled water. The detached BST/PMMA bilayer was fished out on the Si/SiO_2_ wafer, dried at room temperature, treated with acetone to remove PMMA, and annealed at 120 °C for several hours under vacuum conditions to remove residual water. To pattern the BST film into a Hall bar, we employed photolithography. Exposed parts of the film were etched out in HCl/H_2_O_2_/CH_3_COOH aqueous solution. As the top-gate dielectric, 200-nm-thick SiN_*x*_ layer was deposited by using hot-wire CVD at temperatures below 80 °C. The top-gate electrode and metal contact pads were made by Ti/Au deposition.

### Magneto-resistivity measurements

Both AC and DC techniques were employed for resistivity and Hall-effect measurements. The top- and bottom-gate voltages were controlled by two independent Keithley 2450 source meters. A single-axis rotation probe with a capability of mounting the sample horizontally or vertically was used for both out-of-plane and in-plane rotations in magnetic fields. Special care has been taken to isolate the genuine in-plane magnetic-field effects from spurious contributions due to a possible misalignment of the sample (see Supplementary Notes 3 and 4 for details).

### Theoretical calculations

A self-consistent T-matrix approach was employed to calculate the self-energy of the Dirac electrons. The Kubo formula was used to calculate the conductivity within this approximation, including the appropriate vertex corrections (see Supplementary Notes 8 and 9 for details).

### Data availability

The data that support the findings of this study are available from the corresponding author upon request.

## Electronic supplementary material

Supplementary Information
