# Supplementary material for: Planar Hall effect from the surface of topological insulators
Source: Nat Commun. 2017 Nov 7;8:1340. doi: 10.1038/s41467-017-01474-8 (PMC5673905; doi:10.1038/s41467-017-01474-8)
Supplement: Supplementary file 1 — Supplementary Information [file 41467_2017_1474_MOESM1_ESM.pdf]

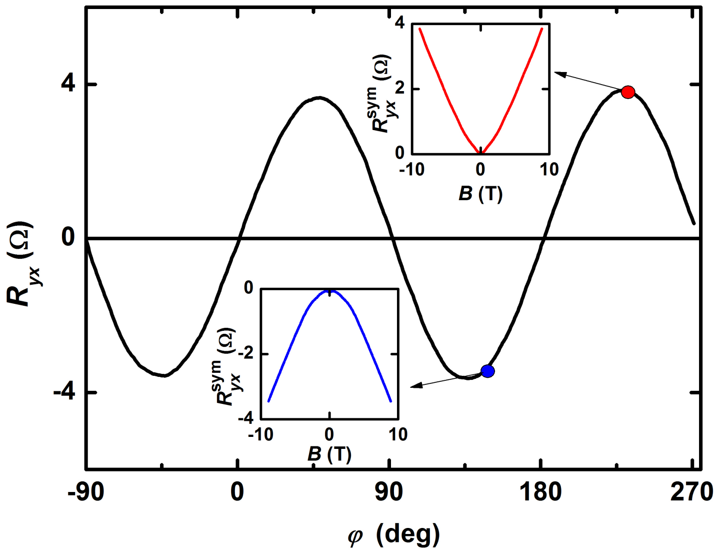

Supplementary Figure 1: **Off-diagonal component of the in-plane magnetoresistance.** Angular dependence of the planar Hall effect measured in one of the 17-nm-thick device at 1.8 K, $B = 9$ T, and $V_{\rm TG} = V_{\rm BG} = 80$ V. φ is the angle between the current and the field directions. Insets show the magnetic-filed dependence of the symmetric part of R_{yx} measured at two different angles φ.

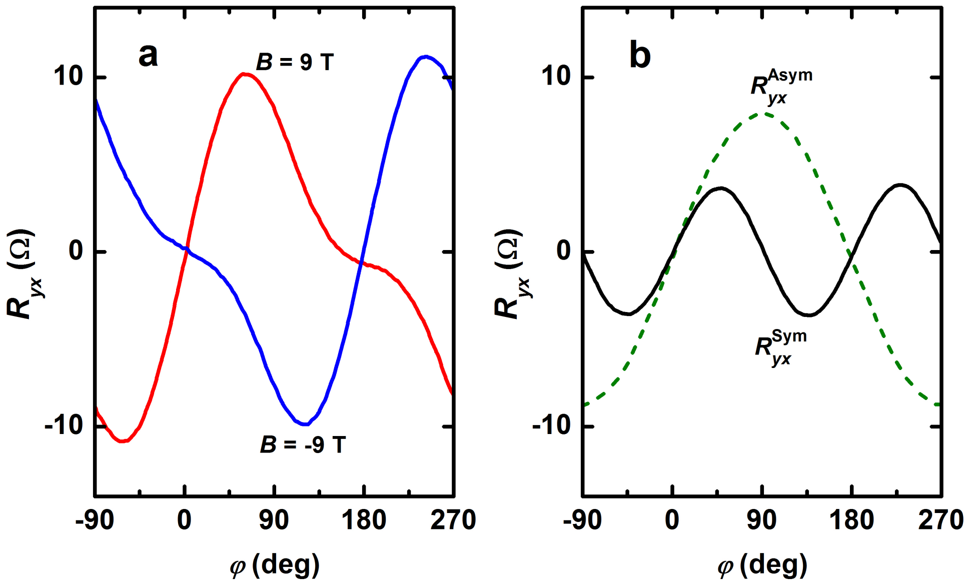

Supplementary Figure 2: **Decomposition of the Planar Hall Effect signal into symmetric and antisymmetric parts.** (**a**) Raw R_{yx} data from a 17-nm device at $\mid B \mid = 9$ T, $V_{\rm TG} = V_{\rm BG} = 80$ V, 1.8 K, and the misalignment of about 1.5°. (**b**) Symmetric and antisymmetric parts of R_{yx} .

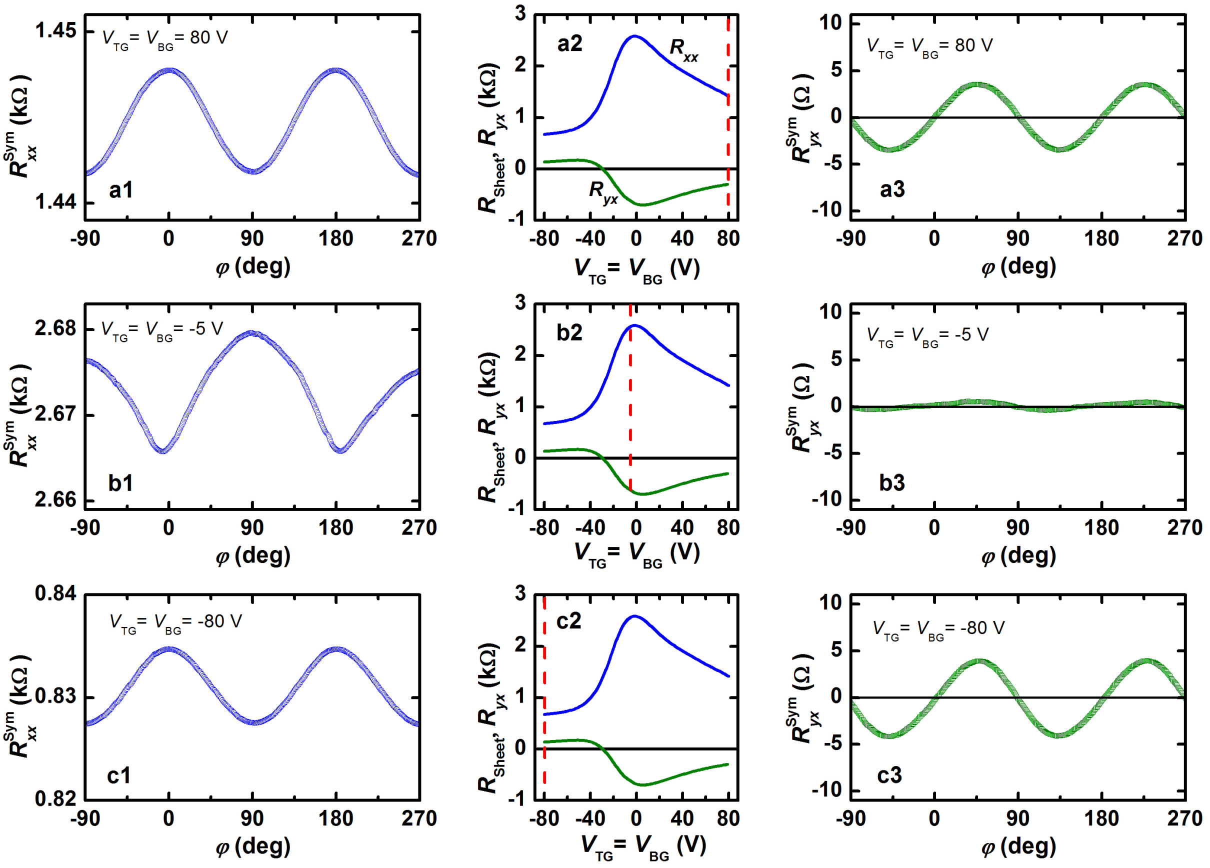

Supplementary Figure 3: **Comparison of the AMR and PHE behaviors at different gate voltages.** (**a1, b1, c1**) $R_{xx}(\varphi)$ in $B = 9$ T. (**a2, b2, c3**) R_{xx}(0T) and R_{yx}(9T) as a function of the gate voltage measured for the out-of-plane configuration of the magnetic field. (**a3, b3, c3**) $R_{yx}^{\rm Sym}(\varphi)$ in $B = 9$ T.

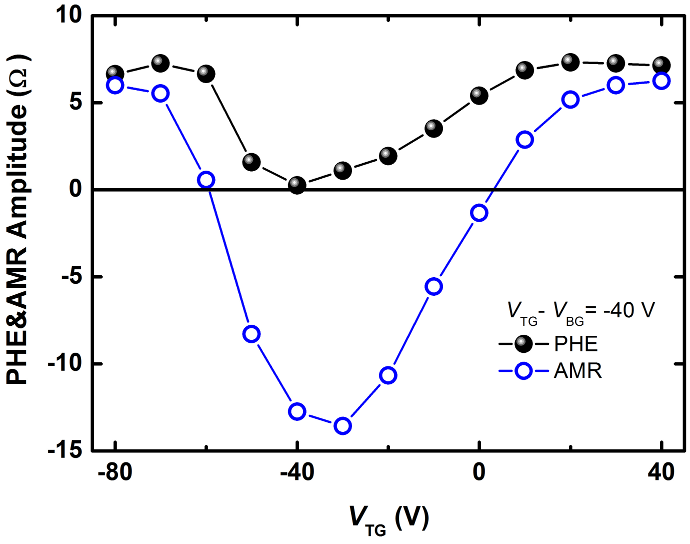

Supplementary Figure 4: **Comparison of the gate-voltage dependences of AMR and PHE amplitudes.** Both amplitudes are measured along the gating path with $V_{\mathrm{TG}} - V_{\mathrm{BG}} = -40$ V in $B = 9$ T at 1.8 K.

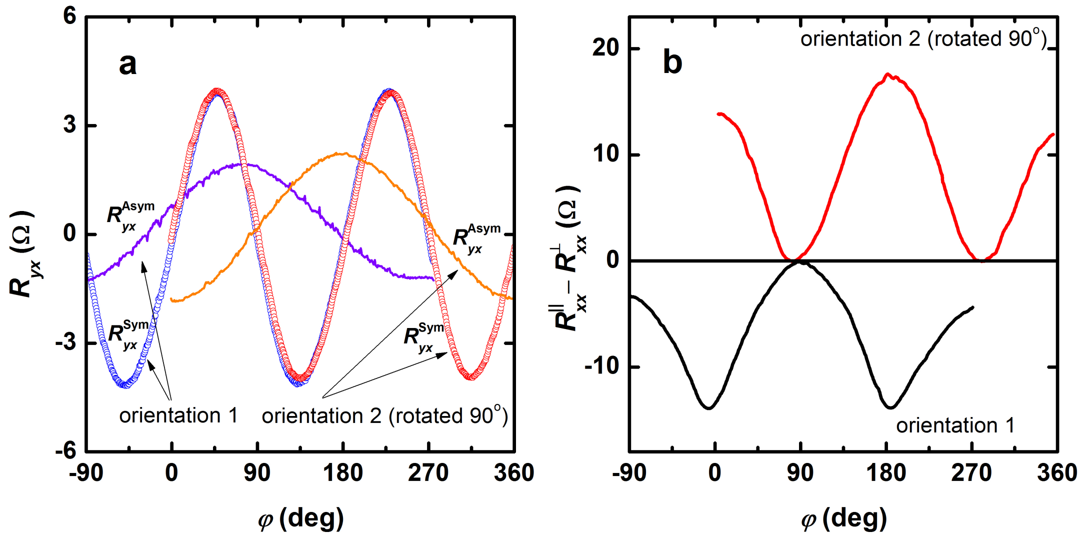

Supplementary Figure 5: **PHE and AMR for different mounting orientations of the sample.** (**a**) PHE in $B = 9$ T, $V_{\mathrm{TG}} - V_{\mathrm{BG}} = -80$ V and (**b**) AMR in $B = 9$ T, $V_{\mathrm{TG}} - V_{\mathrm{BG}} = -5$ V.

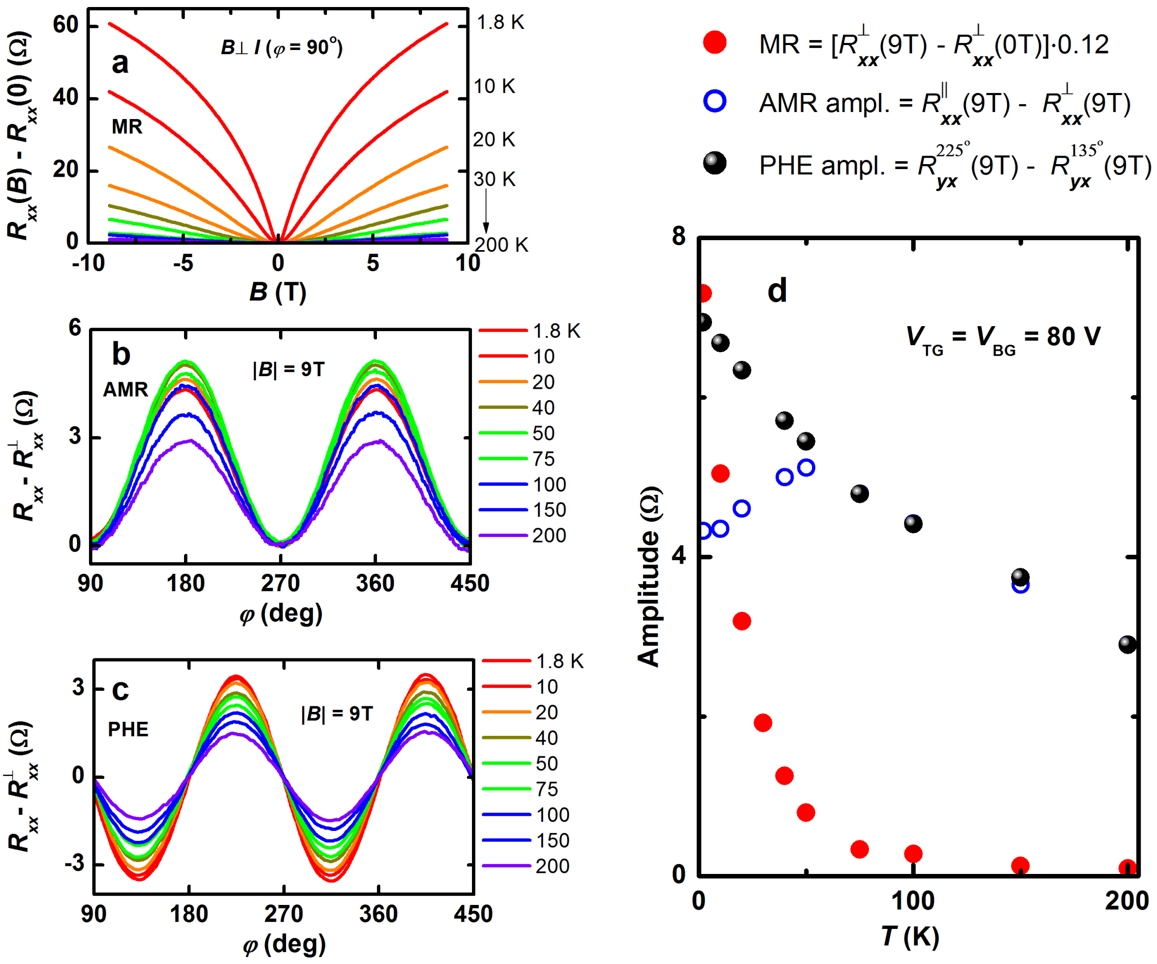

Supplementary Figure 6: **Temperature dependences of MR, AMR, and PHE.** (**a**) MR, (**b**) AMR, (**c**) PHE, and (**d**) their amplitudes in $B = 9$ T. All measuremnets are done on a 17-nm-thick device at $V_{\mathrm{TG}} = V_{\mathrm{BG}} = 80$ V.

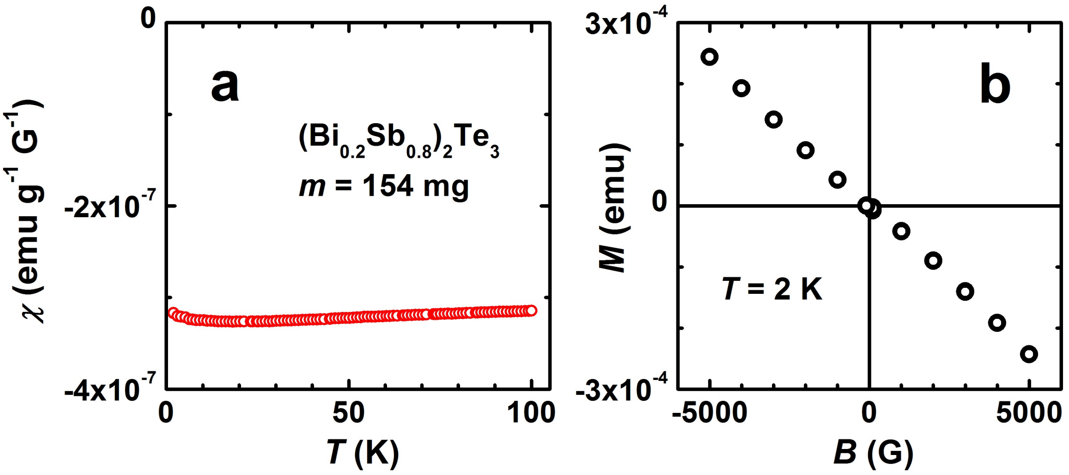

Supplementary Figure 7: **Magnetic properties of $Bi_{0.4}Sb_{1.6}Te_3$ single crystal.** (**a**) The temperature dependence of the mass susceptibility measured in the field of 5000 G. (**b**) M vs. B at 2 K.

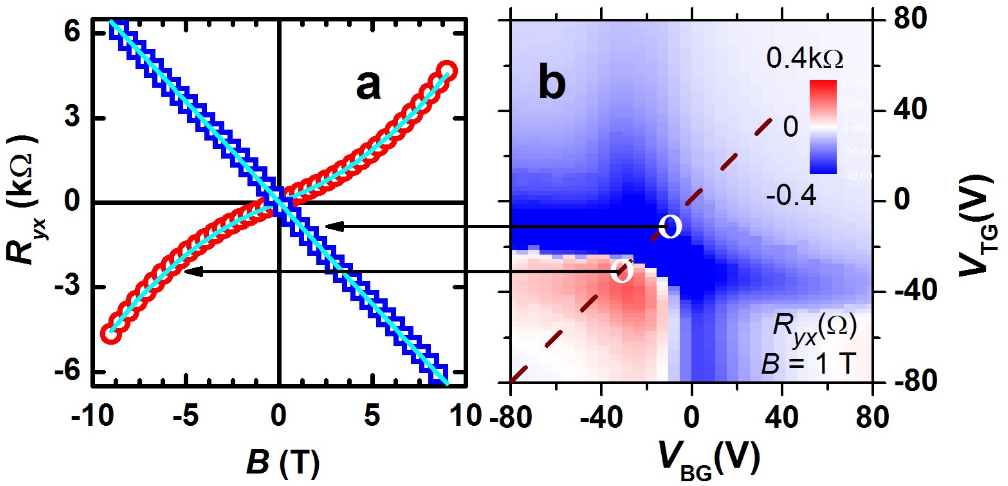

Supplementary Figure 8: **Hall resistance near the Dirac point.** (**a**) $R_{yx}(B)$ measured at $V_{\rm TG} = V_{\rm BG} = -30$ V (open red circles) and -10 V (open blue squares). Solid lines show the fits to the data with a three-band model. (**b**) 2D colour map of $R_{yx}(V_{\rm TG}, V_{\rm BG})$ measured in the out-of-plane magnetic field of 1 T, showing positions where $R_{yx}(B)$ curves were taken.

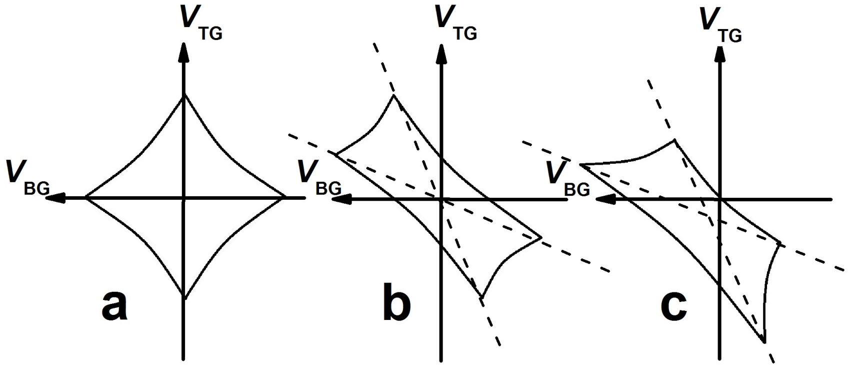

Supplementary Figure 9: **Schematic drawings of the 2D gating map of $\boldsymbol{R}_{xx}(\boldsymbol{V}_{\mathrm{TG}}, \boldsymbol{V}_{\mathrm{BG}})$.** A contour of constant R_{xx} is drawn for the cases of (**a**) independent top and bottom surfaces, (**b**) electrostatically coupled top and bottom surfaces, and (**c**) electrostatically coupled surfaces as in (b) with the admixture of a small contribution of residual bulk n-type carriers.

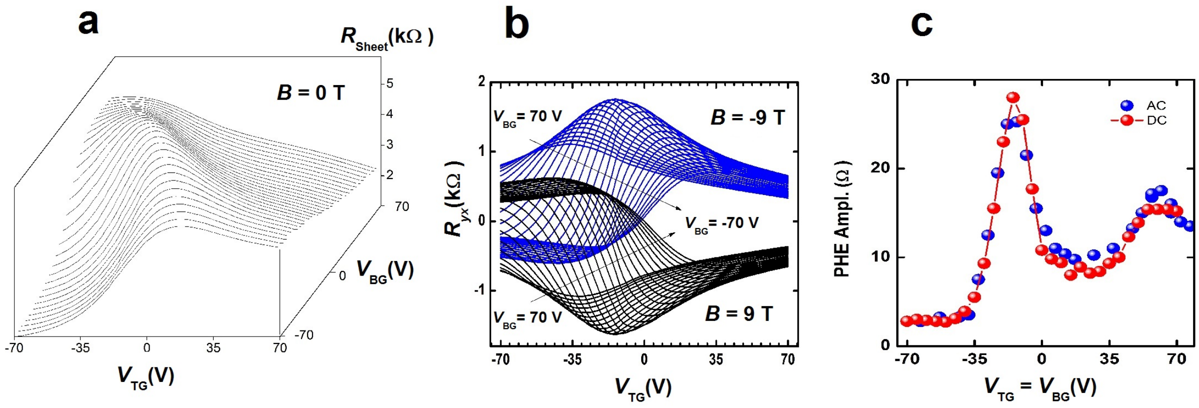

Supplementary Figure 10: **Gating and PHE in the 12-nm-thick device.** (**a**) 3D plot of $R_{xx}(V_{\mathrm{TG}}, V_{\mathrm{BG}})$ measured at $B = 0$ T. (**b**) R_{yx} vs. V_{TG} measured at different V_{BG} in ± 9 T field. (**c**) The PHE amplitude measured along the $V_{\mathrm{TG}} = V_{\mathrm{BG}}$ path in AC and DC measurement setups.

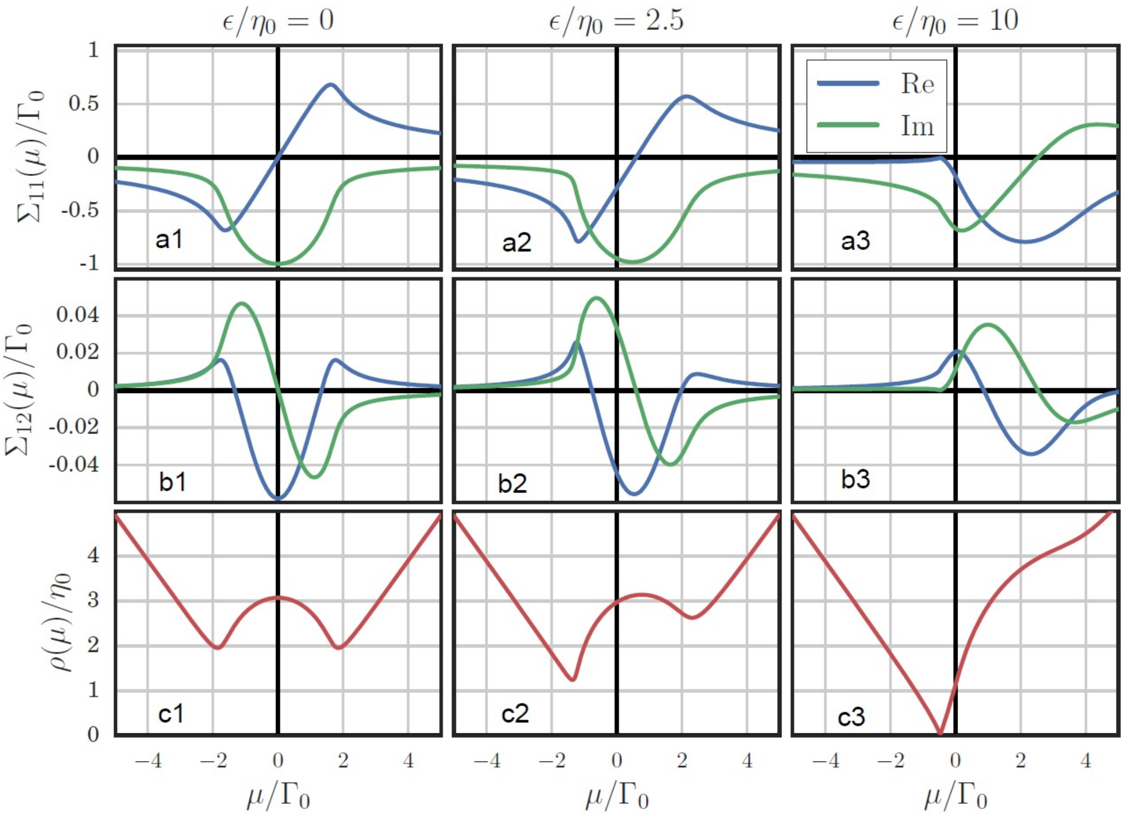

Supplementary Figure 11: **Self energies and density of states as function of the chemical potential and anisotropy $\boldsymbol{\epsilon}$.** (**a1, a2, a3**) The diagonal component of self-energy $\Sigma_{11}(\mu)$ for $\epsilon/\eta_0 = 0,\ 2.5,\ 10$ respectively. (**b1, b2, b3**) The off-diagonal component of self-energy $\Sigma_{12}(\mu) \sim (\Sigma_{11}(\mu))^2$ for $B = 0.25\eta_0$ and the same ϵ parameters. (**c1, c2, c3**) Density of states. Impurity scattering dominates close to the Dirac point which leads to distinct features in all quantities. Parameters: $\Lambda = 10$, $V = \sqrt{4\pi}$.

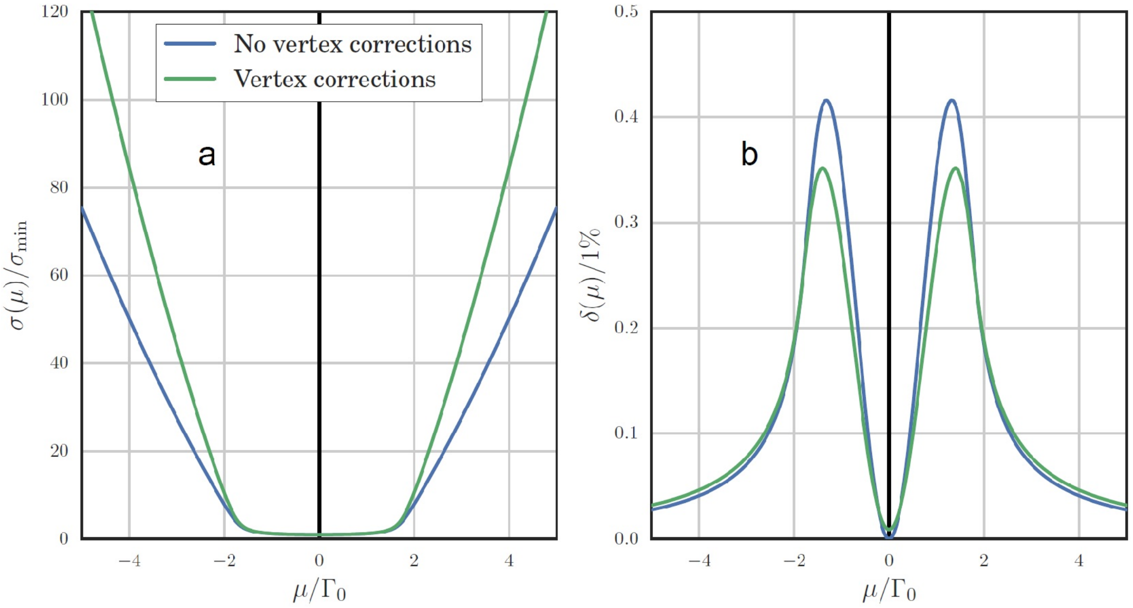

Supplementary Figure 12: **Conductivity for the particle hole symmetric system $\boldsymbol{\epsilon = 0}$.** (**a**) The conductivity with and without vertex corrections. Vertex corrections approximately double the conductivity in the metallic regime but have only a small effect near the peaks of $\delta(\mu)$. (**b**) The dimensionless resistivity anisotropy $\delta(\mu) = (\sigma_{\perp} - \sigma_{\parallel})/\sigma_{\parallel}$ with and without vertex corrections.

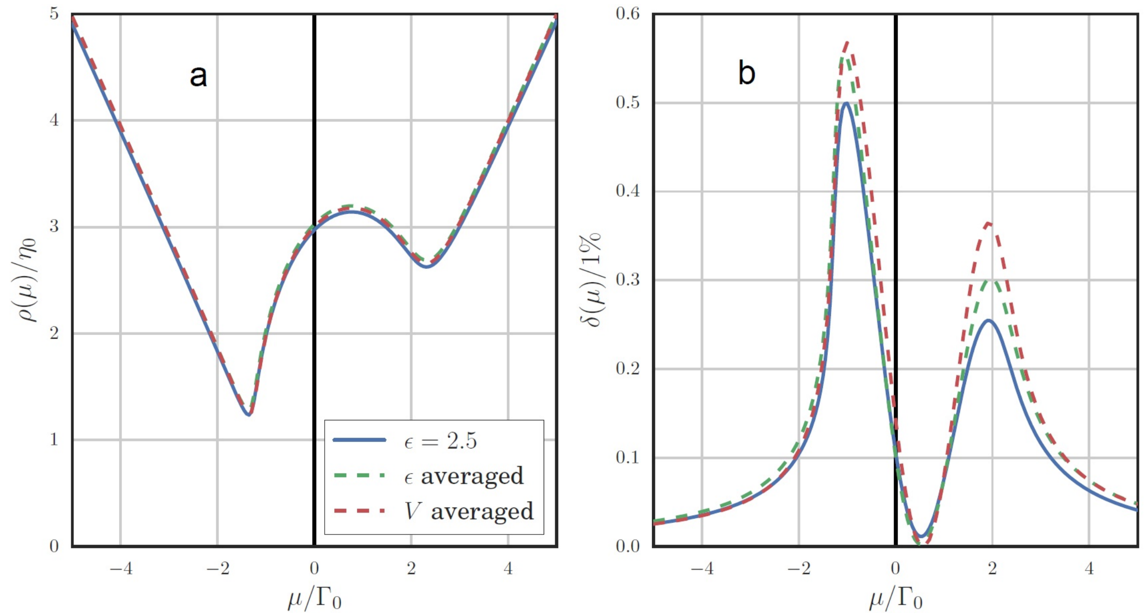

Supplementary Figure 13: **Averaging over impurity parameters.** (**a**) The density of states and (**b**) resistivity anisotropy $\delta(\mu)$ as a function of chemical potential for a single type of impurity with $\epsilon = 2.5\eta_0$, $V = \sqrt{4\pi}$ (blue line), a Gaussian distribution of ϵ values with $\bar{\epsilon} = 2.5\eta_0$ and width $\Delta_\epsilon = \eta_0$ (green, dashed), and a Gaussian distribution of V values with $\bar{V} = \sqrt{4\pi}$ and width $\Delta_V = \bar{V}/8$ (red, dashed). We see that averaging has a negligible effect on the density of states and on the position of the peaks in $\delta(\mu)$. The peaks are, however, slightly broadened and the size of the peaks increases as impurities with smaller ϵ and smaller V get magnetized more strongly, leading to enhanced spin-flip scattering.

Supplementary Note 1: Symmetries and topological protection in the presence of an in-plane magnetic field.

An external in-plane magnetic field breaks time-reversal symmetry and allows for backscattering of electrons at the surface, therefore partially lifting the topological protection of the material. Further crystalline symmetries which exist on average even in a disordered sample can, however, guarantee the existence of gapless surface states for high-symmetry surfaces [1]. In our experiment, we consider the (111) surface with rhombohedral R$\bar{3}$m symmetry. For a field in the [1$\bar{1}$0] direction and equivalent directions obtained by 60° rotations around the surface normal, a mirror symmetry guarantees that no average magnetization is generated perpendicular to the surface. We therefore expect that the system remains gapless for these specific field directions. More precisely, the symmetry is only present on average but also this is sufficient to stabilize a metallic surface state [1].

For other field directions parallel to the surface, by symmetry the formation of a gapful quantum Hall state is possible and expected to happen for $T = 0$ in infinitely large samples. However, for practical purposes this effect is suppressed as the magnetization perpendicular to the surface is (by symmetry) proportional to $B_{\|}^3$. As $B_{\|}$ is smaller than all relevant microscopic energy scales, only a very small effect is expected.

Supplementary Note 2: Angular dependences of AMR and PHE.

When a resistivity anisotropy is induced by an in-plane magnetic field, the resistivity tensor may be written in a diagonalized form by taking the magnetic-field direction as the x' axis of the principal coordinates:

$$\begin{pmatrix} E_{x'} \\ E_{y'} \end{pmatrix} = \begin{pmatrix} R_{\|} & 0 \\ 0 & R_{\perp} \end{pmatrix} \begin{pmatrix} j_{x'} \\ j_{y'} \end{pmatrix}. \tag{1}$$

Here, $E_{x'}$ and $j_{x'}$ are along the magnetic field, and $E_{y'}$ and $j_{y'}$ are perpendicular to the magnetic field. When one transforms this into the coordinate system fixed on the sample, in which x is the current direction and y is the transverse direction on the film plane, the resistivity tensor becomes

$$\begin{aligned} \begin{pmatrix} E_x \\ E_y \end{pmatrix} &= \begin{pmatrix} \cos\varphi & -\sin\varphi \\ \sin\varphi & \cos\varphi \end{pmatrix} \begin{pmatrix} R_{\|} & 0 \\ 0 & R_{\perp} \end{pmatrix} \begin{pmatrix} \cos\varphi & \sin\varphi \\ -\sin\varphi & \cos\varphi \end{pmatrix} \begin{pmatrix} j_x \\ j_y \end{pmatrix}, \\ &= \begin{pmatrix} R_{\|}\cos^2\varphi + R_{\perp}\sin^2\varphi & (R_{\|} - R_{\perp})\cos\varphi\sin\varphi \\ (R_{\|} - R_{\perp})\cos\varphi\sin\varphi & R_{\|}\sin^2\varphi + R_{\perp}\cos^2\varphi \end{pmatrix} \begin{pmatrix} j_x \\ j_y \end{pmatrix}. \end{aligned} \tag{2}$$

By setting $j_y = 0$ as the boundary condition to represent our measurement configuration, one obtains

$$R_{xx} = E_x/j_x = R_{\perp} + (R_{\|} - R_{\perp})\cos^2\varphi, \tag{3}$$

and

$$R_{yx} = E_y/j_x = (R_{\|} - R_{\perp})\cos\varphi\sin\varphi. \tag{4}$$

Here R_{yx} represents the planar Hall effect (PHE), which is essentially an off-diagonal component of the in-plane magnetoresistance (Supplementary Fig. 1). An important difference from the ordinary Hall effect is that this component is symmetric with respect to the magnetic field, as is actually observed in our TI devices (see Supplementary Fig. 1 inset). The anisotropic magnetoresistance (AMR) manifests itself in R_{xx}.

Supplementary Note 3: Spurious contribution to the angular dependence from a sample misalignment .

An arbitrary 3D rotation is specified by an axis of rotation together with an angle of rotation about this axis (one also needs to specify the orientation of the axis and whether the rotation is taken to be clockwise or counterclockwise with respect to this orientation). A counterclockwise rotation about an arbitrary unit vector $\mathbf{u} = (u_x, u_y, u_z)$ by angle ψ is given by the transformation matrix

$$R_{\mathbf{u}}(\psi) = \begin{pmatrix} \cos\psi + u_x^2(1-\cos\psi) & u_x u_y(1-\cos\psi) - u_z \sin\psi & u_x u_z(1-\cos\psi) + u_y \sin\psi \\ u_y u_x(1-\cos\psi) + u_z \sin\psi & \cos\psi + u_y^2(1-\cos\psi) & u_y u_z(1-\cos\psi) - u_x \sin\psi \\ u_z u_x(1-\cos\psi) - u_y \sin\psi & u_z u_y(1-\cos\psi) + u_x \sin\psi & \cos\psi + u_z^2(1-\cos\psi) \end{pmatrix}. \quad (5)$$

A change of the reference frame can be quantified by a rotation about a suitable axis. In this respect, if two frames are related by a rotation about the unit vector $\mathbf{u}$ by angle ψ, a vector $\mathbf{a} = (x, y, z)$ in the original frame is expressed in the new frame as

$$\begin{pmatrix} x' \\ y' \\ z' \end{pmatrix} = R_{\mathbf{u}}(\psi) \begin{pmatrix} x \\ y \\ z \end{pmatrix}. \quad (6)$$

Now we consider the situation of our experiment to rotate the magnetic field in the film plane. In the actual experiment, the magnetic field is fixed in the z axis of the laboratory frame, i.e. $\mathbf{B} = (0, 0, B)$, and the rotation is performed with a mechanical rotator, which rotates the sample around the y axis of the laboratory frame. We assume that, before any rotation (i.e. rotation angle $\psi = 0$), the current is along the x axis of the laboratory frame.

Ideally, for the in-plane rotation, the $x'y'$ plane of the sample frame should be identical to the zx plane of the laboratory frame. In reality, however, there is some misalignment of the sample on the rotator, which results in the deviation of the z' axis of the sample frame from the actual rotation axis (y axis of the laboratory frame). This deviation can be parametrized by using two misalignment angles; namely, rotations of the y axis of the laboratory frame by angles δ and α around the x' and y' axes of the sample frame, respectively. After these two rotations, the y axis of the laboratory frame is brought to the z' axis of the misaligned sample frame.

By using Supplementary Eq. (6), the unit vector of the rotation axis [which is $\mathbf{e}_y = (0, 1, 0)$ in the laboratory frame] is expressed in the sample frame as

$$\mathbf{u}' = \begin{pmatrix} u_{x'} \\ u_{y'} \\ u_{z'} \end{pmatrix} = \begin{pmatrix} \cos\alpha & 0 & -\sin\alpha \\ 0 & 1 & 0 \\ \sin\alpha & 0 & \cos\alpha \end{pmatrix} \begin{pmatrix} 1 & 0 & 0 \\ 0 & \sin\delta & \cos\delta \\ 0 & -\cos\delta & \sin\delta \end{pmatrix} \begin{pmatrix} 0 \\ 1 \\ 0 \end{pmatrix} = \begin{pmatrix} \cos\delta \sin\alpha \\ \sin\delta \\ -\cos\delta \cos\alpha \end{pmatrix}. \quad (7)$$

Also, the magnetic-field vector in the sample frame before the sample rotation ($\psi = 0$) is

$$\mathbf{B}'(0) = \begin{pmatrix} B_{x'}(0) \\ B_{y'}(0) \\ B_{z'}(0) \end{pmatrix} = B \begin{pmatrix} \cos\alpha & 0 & -\sin\alpha \\ 0 & 1 & 0 \\ \sin\alpha & 0 & \cos\alpha \end{pmatrix} \begin{pmatrix} 1 & 0 & 0 \\ 0 & \sin\delta & \cos\delta \\ 0 & -\cos\delta & \sin\delta \end{pmatrix} \begin{pmatrix} 0 \\ 0 \\ 1 \end{pmatrix} = B \begin{pmatrix} -\sin\delta \sin\alpha \\ \cos\delta \\ \sin\delta \cos\alpha \end{pmatrix}. \quad (8)$$

Now, when the sample is rotated clockwise by angle ψ around the axis **u'** obtained in Supplementary Eq. (7), the rotation matrix $R_{\mathbf{u}'}(\psi)$ is

$$\begin{pmatrix} \cos\psi + \cos^2\delta\sin^2\alpha(1-\cos\psi) & \cos\delta\sin\delta\sin\alpha\,(1-\cos\psi) - \cos\delta\cos\alpha\sin\psi & -\cos^2\delta\cos\alpha\sin\alpha\,(1-\cos\psi) - \sin\delta\sin\psi \\ \cos\delta\sin\delta\sin\alpha\,(1-\cos\psi) + \cos\delta\cos\alpha\sin\psi & \cos\psi + \sin^2\delta\,(1-\cos\psi) & -\cos\delta\sin\delta\cos\alpha\,(1-\cos\psi) + \cos\delta\sin\alpha\sin\psi \\ -\cos^2\delta\cos\alpha\sin\alpha\,(1-\cos\psi) + \sin\delta\sin\psi & -\cos\delta\sin\delta\cos\alpha\,(1-\cos\psi) - \cos\delta\sin\alpha\sin\psi & \cos\psi + \cos^2\delta\cos^2\alpha(1-\cos\psi) \end{pmatrix}. \tag{9}$$

Finally, the magnetic-field vector in the sample frame after the sample is rotated clockwise about the **u'** axis by angle ψ is written as

$$\mathbf{B}'(\psi) = \begin{pmatrix} B_{x'}(\psi) \\ B_{y'}(\psi) \\ B_{z'}(\psi) \end{pmatrix} = B\begin{pmatrix} -\cos\alpha\sin\psi\ -\ \sin\delta\sin\alpha\cos\psi \\ \cos\delta\cos\psi \\ \sin\delta\cos\alpha\cos\psi\ -\ \sin\alpha\sin\psi \end{pmatrix}. \tag{10}$$

The magnetic-field component perpendicular to film plane, $B_{z'}(\psi)$, can be written as

$$B_{z'}(\psi) = B\sqrt{\sin^2\delta\cos^2\alpha\ + \sin^2\alpha}\ \cos(\psi\ +\ \phi), \quad \phi = \arctan\left(\frac{\sin\alpha}{\sin\delta\cos\alpha}\right). \tag{11}$$

In our experiment, due to the design of the rotating sample stage, the condition $\alpha \ll \delta \ll 1$ holds and one may obtain

$$B_{z'}(\psi) \simeq B\sin\delta\cos\psi. \tag{12}$$

For B = 9 T and δ as small as 0.5°, the magnetic-field component perpendicular to the sample surface due to the misalignment would be maximally $B\sin\delta \approx 78.5$ mT (785 G). The ordinary orbital MR (expected for magnetic fields perpendicular to the surface) has a quadratic field dependence in this magnetic-field range, and hence its ψ dependence would be $\sim \cos^2\psi$, which is indistinguishable from the AMR behaviour. On the other hand, the ordinary Hall resistivity, caused by a misalignment, has a linear magnetic-filed dependence and has a completely different ψ dependence ($\sim \cos\psi$) than PHE.

Supplementary Note 4: Evidence in the experimental data for sample misalignment.

Supplementary Figure 2a shows the raw R_{yx} data measured in 9 T and −9 T in one of 17-nm-thick devices with an estimated misalignment angel δ of −1.5°. The decomposition of R_{yx} into symmetric and antisymmetric parts is shown in Supplementary Figure 2b. The symmetric part (black solid line in Supplementary Fig. 2b) is the PHE signal, which follows $\sim\cos\varphi\sin\varphi$ dependence. Its amplitude does not depend on a misalignment angle or a mounting configuration. The antisymmetric part (green dashed line) is an ordinary Hall contribution, which here follows $\sim\sin\varphi$ dependence. Its amplitude depends on a misalignment angle δ as $\sim\sin\delta$. The phase is not universal and depends on a mounting configuration as will be shown below.

Supplementary Figure 3 shows the AMR behavior (a1, b1, c1), the gate-voltage dependences of R_{xx}(0T) and R_{yx}(9T) (a2, b2, c2), and the PHE behavior (a3, b3, c3), measured in one of the 17-nm-thick devices with DC current of 30 μA. The AMR and PHE data were taken at three different gate voltages as indicated in the central column by vertical dashed lines. Let us first consider the development of the AMR; it is positive

(i.e. $R_\parallel > R_\perp$) at $V_{\rm TG} = V_{\rm BG} = 80$ V, when the charge carriers on both surfaces are electrons. For $V_{\rm TG} = V_{\rm BG} = -5$ V, when the Fermi level is close to the Dirac point and R_{xx} is maximal, the observed AMR is negative, similar to what was observed in Ref. [2]. Since we have top and bottom gates (and thinner samples), we can move the Fermi level even further into the p-doped side in comparison to the measurements in Ref. [2]. At $V_{\rm TG} = V_{\rm BG} = -80$ V, when the charge carriers on both surfaces are holes, the sign of AMR changed again. If we take a look at the PHE, its amplitude never changes sign (although it becomes close to zero near the Dirac point).

Supplementary Figure 4 shows the gate-voltage dependences of the amplitudes of the AMR and PHE measured along another gating path, in which we kept $V_{\rm TG} - V_{\rm BG} = -40$ V. Here again, the AMR shows a sign change, while the PHE amplitude remains positive.

As has been discussed in Supplementary Note 2, the contribution from the orbital MR due to a misalignment is difficult to distinguish from the genuine AMR signal in the R_{xx} data. Therefore, it is reasonable to assume that the difference in the amplitudes of the AMR and PHE comes from the finite contribution of the orbital MR to AMR, which can give rise to a negative total signal when the MR due to the out-of-plane field is large.

To test this assumption, we performed the following experiment: We measured the same device twice with two different orientations of the sample on the same sample holder. The orientations differ from each other by 90° rotation along the axis perpendicular to the sample surface. The genuine signal should not depend on the orientation of the sample mounting, while for the signal coming from a misalignment, its phase should shift by 90°. As can be seen in Supplementary Figure 5(a), the symmetric parts of R_{yx} (i.e. the genuine PHE signals) were absolutely the same in both measurements. The antisymmetric parts (i.e. ordinary Hall contributions) in the two measurements were indeed shifted by 90° relative to each other. In the results for R_{xx} shown in Supplementary Figure 5b, the phase also shifts by 90° and the amplitude changes from negative to positive, indicating that a major part of the signal is coming from the misalignment and the negative amplitude of the AMR in this case is an artifact.

The temperature dependences of the MR, AMR, and PHE are also useful for distinguishing the genuine signal from spurious contributions. Supplementary Figure 6 shows an example of such an examination, which was made for $V_{TG} = V_{BG} = 80$ V, when both surfaces are filled with electrons. It turns out that the genuine magnetic-field-induced in-plane anisotropy is not very sensitive to temperature, because the amplitude of the PHE decreases only by a factor of two upon raising the temperature from 1.8 to 200 K. On the other hand, the magnitude of the MR in the out-of-plane magnetic field of 9 T drops by a factor of 10 at 50 K [see Supplementary Figure 6(d)]. The temperature dependence of the AMR amplitude is most unusual: It initially increases with increasing temperature, reaches a maximum, and then decreases after merging with the PHE amplitude [Supplementary Figure 6(d)]. A misalignment can easily explain this behaviour: At low temperature, the contribution from the orbital MR (which appears to be negative here) is the largest. This contribution rapidly diminishes with increasing temperature, leading to an apparent increase in the AMR amplitude. At about 50 K, when the spurious contribution from the orbital MR becomes negligible, the AMR amplitude reaches its maximum and becomes identical to the PHE amplitude, as is expected from the resistivity-tensor phenomenology.

The temperature dependence of the AMR amplitude is also useful for examining the relevance of the weak antilocalization (WAL) effect [3]. Although the WAL effect is

primarily considered for magnetic fields applied perpendicular to the film plane [3], it has been proposed that the in-plane magnetic field can still cause a WAL effect when there is a bulk conduction channel [4]. However, one can easily infer that the possible in-plane WAL effect has little to do with the PHE/AMR effect, because the PHE/AMR amplitude decreases only weakly with increasing temperature (Supplementary Fig. 6d). On the other hand, the WAL effect is expected to be diminished quickly with increasing temperature; in particular, one would not expect the WAL effect to be effective at 200 K, at which the PHE/AMR amplitude still keeps about 50% of its size at 1.8 K. In addition, the magnetic-field dependence of the WAL effect [3, 4] is inconsistent with the nearly-B^2 dependence of the PHE/AMR amplitude shown in Fig. 2c of the main text.

Supplementary Note 5: Magnetic properties of BST films.

The origin of the AMR and PHE in BST films could be easily understood if the films were ferromagnetic. To check this possibility, we measured the magnetization of our BST films, using a commercial SQUID magnetometer (Quantum Design MPMS). All of the measured films (with thicknesses ranging from 10 to 140 nm) do not show any magnetization above the sensitivity limit of the instrument ($\sim 10^{-6}$ emu). This can be easily understood by taking into considerations a tiny diamagnetic response expected from $Bi_{2-x}Sb_xTe_3$ and the small amount of the material in a thin film, which is less than 1 μg even for the 140-nm-thick sample. As one can see in Supplementary Figure 7a, the magnetic susceptibility of a large BST single crystal with the mass of 154 mg (and the stoichiometry similar to the films), which we measured along the *ab*-plane, is only about -3×10^{-7} emu g^{-1}G^{-1}. For a relatively large field of 5000 G, the absolute value of the magnetization of the BST crystal reaches about 2.5×10^{-4} emu (Supplementary Fig. 7b), which means that the magnetization of the BST films for similar fields will be less than 10^{-9} emu, *i.e.*, well below the detection limit. On the other hand, it is well known that ferromagnetic BST films, intentionally doped with Cr or V, are easily measurable with a SQUID magnetometer even in samples with a low doping concentration. Therefore, we can exclude the ferromagnetic origin of the AMR and PHE in our BST films.

Supplementary Note 6: Hall resistance across the Dirac point.

While the 2D colour map of the Hall resistance vs. V_{TG} and V_{BG}, measured at a constant magnetic field, gives a good general idea about the type of charge carriers on both top and bottom surfaces, the magnetic-field dependence of R_{yx}, measured at specific gate voltages, can provide useful information on their concentrations. In particular, the value of the residual bulk conductance, which limits the gating efficiency of our devices, is of interest. In order to estimate a residual bulk contribution to the total conductance in our devices, $R_{yx}(B)$ was measured in the out-of-plane field configuration for several gate voltages which can bring the Fermi level across the Dirac points on both surfaces in the 17-nm-thick device (which is discussed in the main text); for example, Supplementary Figure 8a shows $R_{yx}(B)$ measured at $V_{\mathrm{TG}} = V_{\mathrm{BG}} = -10$ V, where the top and bottom surfaces are populated with electrons (open blue squares), and at $V_{\mathrm{TG}} = V_{\mathrm{BG}} = -30$ V, where the top and bottom surfaces are populated with holes (open red circles). The positions on the 2D gating map, where these two curves were measured, are shown in Supplementary Figure 8b. The change in the type of carriers across the Dirac points on

the two surfaces is obvious from the change in the sign of the $R_{yx}(B)$ slope. Moreover, the specific shape of the $R_{yx}(B)$ curve at $V_{\rm TG} = V_{\rm BG} = -30$ V, showing a steeper slope at higher fields, is an indication of the presence of n-type carriers with a concentration smaller than the concentration of p-type carriers. Fitting with a three-band model (top surface, bottom surface, and a bulk channel), shown by solid lines in Supplementary Figure 8a, gives the concentration of bulk electrons $N_{\rm 3D} = 6\times10^{16}$ cm^{-3}, which for a 17-nm-thick film is equivalent to $\sim 10^{11}$ cm^{-2}. We assume that this bulk contribution is the same for different gate voltages, at least when the Fermi level is near the Dirac points. For the surface charge carriers, the concentration changes from $n_{\rm b} = 2\times10^{11}$ cm^{-2} and $n_{\rm t} = 5.9\times10^{11}$ cm^{-2} on the electron side to $p_{\rm b} = 7.5\times10^{11}$ cm^{-2} and $p_{\rm t} = 2\times10^{11}$ cm^{-2} on the hole side, where subscripts b and t refer to the bottom and top surfaces, respectively. (Note that the measurement of a series of $R_{yx}(B)$ curves at several gate voltages makes the three-band analysis to be much more reliable than an arbitrary three-band fitting to a single curve). Since the total change in the surface carrier concentration can exceed $\pm\ 3\times10^{12}$ cm^{-2} at the highest gate voltages, we can conclude that the transport in our devices is surface dominated.

In addition, the above analysis yields the information of the carrier mobility for each channel. It is useful to note that the obtained carrier mobility of the top and bottom surfaces were 1400 and 900 cm^2/Vs, respectively, near the Dirac point. These values are essentially the same as those of pristine BST films, which evinces that our gate fabrication process causes no noticeable degradation in the surface states. The carrier mobility can also be estimated in a simpler way, just from the sheet resistance and the Hall coefficient: In our dual-gate device, the low-temperature sheet resistance $R_{\rm xx}$ was 2.73 kΩ (Fig. 1a) and the Hall coefficient $R_{\rm H}$ was 2.8×10^6 cm^2/C at zero gate voltage (Fig. 2d); these values give an estimate of the mobility $\mu = R_{\rm H}/R_{\rm xx} = 1000$ cm^2/Vs, which is consistent with the more elaborate estimate of the mobility shown above.

A small contribution of the residual bulk electrons to the transport can be also seen in the 2D gating map of $R_{xx}(V_{\rm TG}, V_{\rm BG})$ (Fig. 1d in the main text). A schematic of the development of such 2D map is shown in Supplementary Figure 9. In the ideal situation, when two surfaces can be gated independently, the horizontal axis in Supplementary Figure 9a would mark the crossing from electrons to holes on the top surface, while the vertical axis would correspond to the crossing from electrons to holes on the bottom surface. Both axes go through the charge neutrality point (CNP). A contour of a constant R_{xx} in this case would have a broadened "+" (diamond) shape centered at CNP (Supplementary Figure 9a). If the two surfaces cannot be gated independently, which means that the top gate will change the concentration of carriers not only on the top surface, but also somewhat on the bottom surface (and similarly for the bottom gate), then instead of the horizontal and vertical axes to mark the position of the crossing from electrons to holes, one will find two lines, inclined to each other at some angle (see, for example, Fig. 1d in Ref. [5]). This will lead to the rotation and some distortion of the contour in the 2D map (Supplementary Figure 9b). The residual bulk contribution (even when it is small) will further distort the shape of the contour as shown in Supplementary Figure 9c. This is what we observe in our dual-gated device as can be seen in Fig. 1d in the main text. The residual bulk n-type carriers shift the boundaries of the diamond in the direction of the left lower corner, which means that to reach the same level of the resistance as in the ideal case, one must apply a higher negative voltage.

The change of the carrier type on the two surfaces can be also seen in a distorted "+" shape of the 2D map of $R_{yx}(V_{\rm TG}, V_{\rm BG})$ (Figs. 1f and 2e in the main text). In low

fields, the four regions, corresponding to the different types of charge carriers on the two surfaces as schematically shown in Supplementary Figure 8a, can be clearly distinguished (Supplementary Fig. 8b). The shape of constant $R_{yx}(V_{\mathrm{TG}}, V_{\mathrm{BG}})$ contours on the 2D map, which depends not only on the concentrations of carries on both surfaces, but also on their mobilities, is dominated by the crossing of the Dirac points on the top and bottom surfaces. Similar behaviour of the $R_{yx}(V_{\mathrm{TG}}, V_{\mathrm{BG}})$ has been also reported for a dual-gated insulating BSTS flake in Ref. [5].

Supplementary Note 7: 12-nm-thick device.

Supplementary Figure 10 shows the data obtained on another dual-gated device, which was made using a 12-nm-thick BST film. As can be seen from Supplementary Figures 10a and 10b, the gating behaviour of this device is qualitatively the same as the behaviour of the 17-nm-thick device shown in the main text: The type of charge carriers can be changed from the n- to p-type through the Dirac points on both surfaces. Accordingly, the PHE amplitude as a function of the gate voltage along the $V_{\mathrm{TG}} = V_{\mathrm{BG}}$ path shows two pronounced peaks and a minimum near the Dirac point as we observed in the 17-nm-thick device.

Supplementary Note 8: Self-consistent T-matrix approximation.

To model scattering from a random magnetic field we consider two-dimensional Dirac electrons coupled to impurities located at random positions $\mathbf{R}_i$ with the density n^{imp} (as in the main text):

$$H = \sum_{\mathbf{k},\alpha,\beta} h_{\alpha\beta}(\mathbf{k})\psi^\dagger_\alpha(\mathbf{k})\psi_\beta(\mathbf{k}) + \sum_{\alpha,\beta}((\epsilon-\mu)\delta_{\alpha\beta} - \mathbf{B}\cdot\boldsymbol{\sigma})d^\dagger_\alpha d_\beta + V\sum_{\mathbf{k},\alpha,i} e^{-i\mathbf{k}\cdot\mathbf{R_i}}\psi^\dagger_\alpha(\mathbf{k})d_\alpha + \text{h.c.}, \tag{13}$$

where

$$h_{\alpha\beta}(\mathbf{k}) = v_{\mathrm{F}}(k_x\sigma_y - k'_y\sigma_x)_{\alpha\beta}, \tag{14}$$

is the (momentum shifted) Hamiltonian of the free Dirac fermions on the surface of the topological insulator.

From this we can identify the Green's function for the Dirac electrons

$$G^{\mathrm{D}}_{\alpha\beta}(\omega) = (\omega + \mu - \Sigma(\omega) - h(\mathbf{k}))^{-1}_{\alpha\beta}, \tag{15}$$

where the self-energy matrix $\Sigma(\omega)$ is due to scattering from the impurities which have a Green's function (T-matrix)

$$T(\omega) = V^2 g^{\mathrm{imp}}_{\alpha\beta}(\omega) = V^2(\omega + \mu - \epsilon - \Delta(\omega) + g\mu_{\mathrm{B}}[\mathbf{B}\cdot\boldsymbol{\sigma}])^{-1}_{\alpha\beta}, \tag{16}$$

where $\Delta(\omega)$ describes the hybridization of the impurity state with the continuum of Dirac electrons. G^{D}, g^{imp}, Σ, and Δ are all 2×2 matrices. When no magnetization of the impurity is present (i.e. $B = 0$), the self-energy $\Sigma(\omega)$ and hybridization $\Delta(\omega)$ are diagonal in spin-space.

To calculate the self-energy and hybridization function appearing in Supplementary Eqs. (15) and (16) we take the first order of an n^{imp} expansion of these quantities. This

corresponds to scattering events arising from only a single impurity. Hence, diagrammatically (see below), the irreducible contributions involve only impurity lines from a single scattering center and this approximation is known as the self-consistent T-matrix approximation (SCTMA) [8]. The SCTMA becomes mathematically exact in the limit that the ratio of the density of impurities $n^{\rm imp}$ to the density of electron states $\rho(\mu)$ becomes zero. This condition is not satisfied near the Dirac point where the density of states of the clean system is zero and so the SCTMA is not rigorously valid here. Despite this it has been shown that such an approximation accurately captures the qualitative physics of the a Dirac system coupled to impurities in the metallic regime far from the Dirac as well as in the impurity dominated regime close to the Dirac point [6].

Hence within the SCTMA the self-energy is given by

$$\Sigma_{\alpha\beta}(\omega) = n^{\rm imp}$$

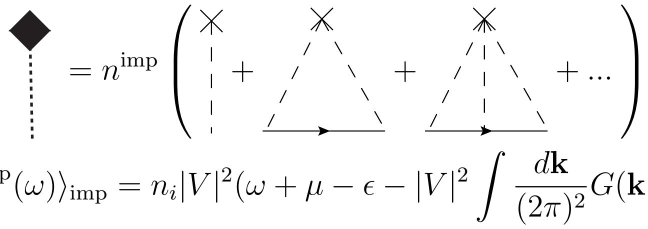

$$= n^{\rm imp}|V|^2\langle g^{\rm imp}_{\alpha\beta}(\omega)\rangle_{\rm imp} = n_i|V|^2\left(\omega+\mu-\epsilon-|V|^2\int\frac{d\mathbf{k}}{(2\pi)^2}G(\mathbf{k},\omega)+g\mu_B[\mathbf{B}\cdot\boldsymbol{\sigma}]\right)^{-1}_{\alpha\beta}, \tag{17}$$

where the average $\langle . \rangle_{\rm imp}$ is over the (random) positions of impurities and the hybridization matrix is given by

$$\Delta_{\alpha\beta}(\omega) = |V|^2\int\frac{d\mathbf{k}}{(2\pi)^2}G^{\rm D}_{\alpha\beta}(\omega,\mathbf{k}). \tag{18}$$

The Dirac Green's function $G(\mathbf{k},\omega)$ appearing in the hybridization function of $\langle g^{\rm imp}_{\alpha\beta}(\omega)\rangle_{\rm imp}$ includes $\Sigma(\omega)$ and so Supplementary Eq. (17) is a self-consistent equation for the self-energy. For non-zero fields Supplementary Eq. (17) is a 2×2 matrix equation.

When $B = 0$ the angular integral over the off-diagonal components of the Dirac Green's function cancels in the self-consistent equation Supplementary Eq. (17), and the off-diagonal component of self-energy Σ_{12} becomes zero. The remaining diagonal element $\Sigma_{11} = \Sigma_{22}$ is then given by [6]

$$\begin{aligned}\Sigma_{11}(\omega) &= n^{imp}|V|^2\left(\omega+\mu-\epsilon-|V|^2\int_0^\Lambda\frac{dk}{2\pi}\frac{\omega+\mu-i\delta-\Sigma(\omega)}{(\omega+\mu-i\delta-\Sigma(\omega))^2-v_f^2k^2}\right)^{-1}\\ &\approx n^{imp}|V|^2\left(\omega+\mu-\epsilon+\frac{|V|^2}{4\pi v_f^2}(\omega+\mu-\Sigma(\omega))\ln\left(\frac{-\Lambda^2}{(\omega+\mu-\Sigma(\omega))^2}\right)\right)^{-1},\end{aligned} \tag{19}$$

where we have introduced a cut-off Λ due to the logarithmic behavior of the integral. At zero frequency the equation has two distinct regimes: (i) In the metallic regime, where $|\mu|$ is large, both real and imaginary parts of self-energy are small, $\Sigma \sim 1/\mu$. (ii) An impurity dominated regime near the Dirac point, where the self-consistency becomes important.

Three examples of real and imaginary part of the self-energy and spectral functions are shown in Supplementary Figure 11. The impurity dominated regime is characterized by a large increase in the absolute value of the imaginary part of the self-energy; correspondingly, due to Kramers-Krönig relation, there are two maxima in the real part of the self-energy. These peaks are associated with dips in the spectral functions.

For the particle-hole symmetric situation, $\epsilon = 0$, Supplementary Eq. (19) is purely imaginary at the center of the impurity dominated regime at $\mu = 0$. The energy scale

here, $\Sigma(\mu = 0) = -i\Gamma_0$, defines the width of this impurity dominated regime. From Supplementary Eq. (19) we see that Γ_0 is given by self-consistently solving

$$\Gamma_0 = \sqrt{\frac{2\pi n^{\text{imp}} v_f^2}{\ln\left(\frac{\Lambda}{\Gamma_0}\right)}}. \tag{20}$$

For our discussion it is important to distinguish between weakly and strongly scattering impurities. Within our model, strong impurity scattering is realized for small ϵ, when scattering is approximately resonant. An inspection of the denominator in Supplementary Eq. (19) reveals that strong, approximately resonant impurity scattering is realized for $|\epsilon| \lesssim |V|^2\Gamma_0^2/(4\pi v_f^2) = \eta_0$. In contrast, when $\epsilon \gg \eta_0$, scattering from off-resonance impurities is weak.

The density of states is given by the trace of the Dirac Green's function at zero frequency,

$$\rho(\mu) = -\frac{1}{\pi}\text{Im}\left\{\text{Tr}\int \frac{d^2k}{(2\pi)^2} G(\mu, \mathbf{k})\right\} = \text{Im}\left\{\frac{2n^{\text{imp}}|V|^2}{\pi\Sigma(\mu)}\right\}, \tag{21}$$

where the second line can be obtained by inserting the self-consistent equation Supplementary Eq. (19) solved for the hybridization function. The density of states is also shown in Supplementary Figure 11 for the same resonance values. From this we see that in the metallic regime the density of states is linear, as in the clean system. However close to the Dirac point the density of states is strongly affected by the presence of impurities with new states created between the bounds of the regime set by the energy scale Γ_0.

At finite $\mathbf{B}$ (taking the $\|$-direction as the x-direction) the self-consistent T-matrix equation, Supplementary Eq. (17), becomes a full matrix equation with non-zero off-diagonals of the self-energy matrix $\Sigma(\omega)$ and hybridization matrix $\Delta(\omega)$. This matrix equation is now

$$\begin{aligned}\Sigma(\omega) &= n_i|V|^2 \begin{pmatrix} \omega + \mu - \Delta_{11}(\omega) - \epsilon & g\mu_{\text{B}}B - \Delta_{12}(\omega) \\ g\mu_{\text{B}}B - \Delta_{12}(\omega) & \omega + \mu - \Delta_{11}(\omega) - \epsilon \end{pmatrix}^{-1} \\ &\approx \Sigma_0(\omega)\mathbb{1} + g\mu_{\text{B}}B\frac{\Sigma_0(\omega)^2}{n_{\text{imp}}|V|^2}\sigma_x,\end{aligned} \tag{22}$$

where the last line is valid for small $\mathbf{B}$ and $\Sigma_0(\omega)$ is defined using the zero-field self-consistent equation

$$\Sigma_0(\omega) = \frac{n_i|V|^2}{\omega + \mu - \epsilon - \Delta_{11}(\omega)}. \tag{23}$$

Examples of the real and imaginary part of $\Sigma_{12}(\omega)$ are shown in Supplementary Figure 11.

As discussed in the main text (see discussion below Supplementary Eq. (2) there), our main experimental finding, the two-peak structure in the anisotropic magnetoresistance, can be traced back to the second line in Supplementary Eq. (22): $\text{Im}\Sigma_{12}(\mu)$ is proportional to $\text{Im}[\Sigma_{11}(\mu)^2] = 2\text{Im}\Sigma_{11}\text{Re}\Sigma_{11}$. Ultimately, this implies that the peaks in $\text{Re}\Sigma_{11}$ lead to peaks in the gate-voltage dependence of the anisotropy, see main text.

Note, however, that the two peaks in $\text{Im}\Sigma_{12}(\mu)$ and the related two-peak structure found in the conductivity (see main text) will vanish in a regime where all impurities are weakly scattering. In this Born limit (reached nominally in our model for $\epsilon \gg \eta_0$), where the impurity dominated regime is exponentially suppressed, $\Sigma_{12}(\mu)$ is proportional to $\rho(\mu)$ and no peaks will be visible.

Supplementary Note 9: Conductivity within SCTMA.

The DC conductivity is given by the Kubo formula [8]

$$\sigma^{\alpha\beta}(\mu) = \lim_{\Omega\to 0}\frac{1}{\Omega}\int\frac{d^2\mathbf{k}}{(2\pi)^2}\int_0^\infty dt\; e^{i\Omega t}\; Tr\langle[\hat{J}^\alpha(t),\hat{J}^\beta(0)]\rangle, \tag{24}$$

where the current operator for spin-momentum locked surface states is given by $\mathbf{J}{=}e\frac{\partial H}{\partial\mathbf{k}} = ev_{\mathrm{F}}\,(\sigma_y, -\sigma_x)$. Ignoring for the moment vertex corrections (see below), the conductivity at $T=0$ is given by

$$\begin{aligned}\sigma_0^{\alpha\beta}(\mu) &= \lim_{\Omega\to 0}\mathrm{Im}\left\{\frac{n_{\mathrm{F}}(\omega+\Omega)-n_{\mathrm{F}}(\omega)}{\Omega}\Pi_0(\omega+\Omega)\right\}\\ &= -\mathrm{Im}\left\{\int\frac{d\omega}{i\pi}\frac{\partial n_{\mathrm{F}}(\omega)}{\partial\omega}\int\frac{d^2\mathbf{k}}{(2\pi)^2}\mathrm{Tr}\langle\hat{J}^\alpha G(\mathbf{k},\omega)\hat{J}^\beta G^\dagger(\mathbf{k},\omega)\rangle\right\}\\ &= -\mathrm{Im}\left\{\frac{e^2v_{\mathrm{F}}^2}{i\pi}\int\frac{d^2\mathbf{k}}{(2\pi)^2}\mathrm{Tr}\langle\sigma^\alpha G(\mathbf{k},\mu)\sigma^\beta G^\dagger(\mathbf{k},\mu)\rangle\right\}.\end{aligned} \tag{25}$$

Additional terms of the form $\langle\hat{J}^\alpha G\hat{J}^\beta G\rangle$ and $\langle\hat{J}^\alpha G^\dagger\hat{J}^\beta G^\dagger\rangle$ will be present but are equal for $\sigma^\parallel$ and $\sigma^\perp$ and so do not contribute to the anisotropy in MR. This is because $\mathrm{Im}\Sigma_{12}$ always has the same sign for these terms and so can be completely eliminated by a shift in k_y. In all three types of terms $\mathrm{Re}\Sigma_{12}$ can also be eliminated in a similar manner and so does not contribute to the AMR. The resulting difference in conductivity can be interpreted in terms of the ratio between spin-flip and non-spin-flip scattering (see main text).

For the particle-hole symmetric system (i.e. $\epsilon = 0$), the conductivity at the Dirac point within the SCTMA is $e^2/2\pi^2$. This is a quarter the value found in graphene (which has an additional two valley and spin degrees of freedom) within the same approximation [6].

The vertex corrections to the conductivity of graphene vanish for short-ranged impurities [6]. The locking of spin and momentum for surface states of topological insulators implies that impurity scattering is always angular dependent. This implies that vertex corrections do not vanish in this case and have to be taken into account within the self-consistent T-matrix approximation. Within the SCTMA framework, vertex corrections are obtained from a sum of ladder diagrams [8, 6],

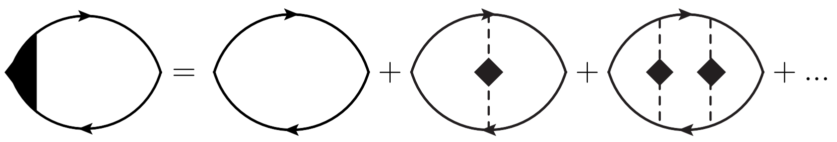

(26)

Fortunately, it is not necessary to solve an integral equation to resum the vertex corrections within our model. Instead, one can use the following trick: All $\mathbf{k}$ summations can directly be done by defining the 4×4 matrices

$$\mathcal{M}(\omega,\Omega) = \int\frac{d^2\mathbf{k}}{(2\pi)^2}G(\mathbf{k},\omega+\Omega)\otimes G^\dagger(\mathbf{k},\omega) \tag{27}$$

and

$$\mathcal{T}(\omega,\Omega) = T(\omega+\Omega)\otimes T(\omega), \tag{28}$$

where $\otimes$ is the Kronecker product. Within this space the Pauli-matrices in the current vertex map to column vectors and Supplementary Eq. (26) can be written in terms of a geometric series of 4×4 matrices

$$\begin{aligned}\sigma^{\alpha\beta}(\mu) &= \lim_{\Omega\to 0}\mathrm{Re}\left\{e^2 v_{\mathrm{F}}^2\int\frac{d\omega}{\pi}\frac{\Delta n_{\mathrm{F}}(\omega)}{\Omega}\sigma^{\alpha}.(\mathcal{M}+n^{\mathrm{imp}}\mathcal{M}\mathcal{T}\mathcal{M}+...).\sigma^{\beta}\right\}\\ &= \lim_{\Omega\to 0}\mathrm{Re}\left\{e^2 v_{\mathrm{F}}^2\int\frac{d\omega}{\pi}\frac{\Delta n_{\mathrm{F}}(\omega)}{\Omega}\sigma^{\alpha}.(\mathcal{M}.(\mathbb{1}_4 - n^{\mathrm{imp}}\mathcal{T}\mathcal{M})^{-1}).\sigma^{\beta}\right\}.\end{aligned} \tag{29}$$

To be precise, the formulas given above are only complete when one calculates the anisotropy of the resistivity, $\sigma_\perp - \sigma_\parallel$. Otherwise one has also to include extra isotropic contributions arising from contributions where either $G^\dagger$ is replaced by G or G by $G^\dagger$, see discussion below Supplementary Eq. (25).

We would like to emphasize that the limit $\Omega\to 0$ has to be taken with some care (i.e. only at the very end of the calculation) in this Dirac system. This is related to the fact that for finite magnetization $\langle \hat{J}^x\rangle(\mu)\neq 0$ even for vanishing electric field.

As can be seen from Supplementary Figure 12, the vertex corrections approximately double the conductivity in the metallic regime far from the Dirac point, but for the ratio $\delta(\mu) = (\sigma_\perp - \sigma_\parallel)/\sigma_\parallel$ the vertex corrections cause only a small reduction in the vicinity of the peaks.

In reality the topological insulator surface may contain different types of impurities described, for example, by a distribution of parameters V and ϵ. To check the robustness of our description of the experiment, we therefore show in the following that such distributions do not affect our conclusions.

Within the SCTMA, the average over parameters can directly be implemented by averaging over ϵ and/or V in Supplementary Eq. (17). To perform this averaging we assume that the distribution of parameters is described by a Gaussion distribution $n(\epsilon, V)$ with averages $\bar{\epsilon}$ and $\bar{V}$, widths Δ_ϵ and Δ_V, and $\int n(\epsilon,V)\,d\epsilon\,dV = n^{\mathrm{imp}}$. To calculate vertex corrections, Supplementary Eq. (28) has to be replaced by

$$\mathcal{T}(\omega,\Omega) = \int d\epsilon\,dV\,\frac{n(\epsilon,V)}{n^{\mathrm{imp}}}T(\omega+\Omega)\otimes T(\omega). \tag{30}$$

Supplementary Figure 13 shows the results of both V and ϵ averaging. We see that the distribution has no effect on the position of the peaks in $\delta(\mu)$, which are only slightly broadened. The height of the peaks in increased due to the enhanced magnetic scattering arising from impurities with smaller ϵ and V. Most importantly, averaging over impurity distributions does not affect our interpretation of the anisotropic magnetoresistance put forward in the main text.

Supplementary References

[1] Chiu, C.-K., Jeffrey C.Y. Teo, J. C. Y., Schnyder, A. P. & Ryu S. Classification of topological quantum matter with symmetries. *Rev. Mod. Phys.* **88**, 035005 (2016).

[2] Sulaev, A. *et al.* Electrically tunable in-plane anisotropic magnetoresistance in topological insulator $BiSbTeSe_2$ nanodevices. *Nano Lett.* **15**, 2061-2066 (2015).

[3] Chen, J. *et al.* Gate-Voltage Control of Chemical Potential and Weak Antilocalization in Bi_2Se_3. *Phys. Rev. Lett.* **105**, 176602 (2010).

[4] Lin, C. J. *et al.* Parallel field magnetoresistance in topological insulator thin films. *Phys. Rev. B* **88**, 041307 (2013).

[5] Xu, Y., Miotkowski, M., & Chen, Y. Quantum transport of two-species Dirac fermions in dual-gated three-dimensional topological insulators. *Nat. Commun.* **7**, 11434 (2016).

[6] Ostrovsky, P. M., Gornyi, I. V. & Mirlin, A. D. Electron transport in disordered graphene. *Phys. Rev. B* **74**, 235443 (2006).

[7] Löfwander, T. & Fogelström, M. Impurity scattering and Motts formula in graphene. *Phys. Rev. B* **76**, 193401 (2007).

[8] Mahan, G. D. Many-particle physics. 3rd ed. (Springer, 2000).
